# Mitochondrial Cochaperone Mge1 Is Involved in Regulating Susceptibility to Fluconazole in *Saccharomyces cerevisiae* and *Candida* Species

**DOI:** 10.1128/mBio.00201-17

**Published:** 2017-07-18

**Authors:** Liesbeth Demuyser, Erwin Swinnen, Alessandro Fiori, Beatriz Herrera-Malaver, Kevin Verstrepen, Patrick Van Dijck

**Affiliations:** a VIB-KU Leuven Center for Microbiology, Flanders, Belgium; b Laboratory of Molecular Cell Biology, Institute of Botany and Microbiology, KU Leuven, Leuven, Belgium; c Laboratory of Functional Biology, Institute of Botany and Microbiology, KU Leuven, Leuven, Belgium; d Laboratory for Genetics and Genomics, KU Leuven, Leuven, Belgium; Duke University Medical Center

**Keywords:** *Candida albicans*, *Candida glabrata*, Fe-S cluster, Mge1, *Saccharomyces cerevisiae*, antifungal susceptibility, fluconazole, iron metabolism, mitochondrial chaperone

## Abstract

*MGE1* encodes a yeast chaperone involved in Fe-S cluster metabolism and protein import into the mitochondria. In this study, we identified *MGE1* as a multicopy suppressor of susceptibility to the antifungal fluconazole in the model yeast *Saccharomyces cerevisiae*. We demonstrate that this phenomenon is not exclusively dependent on the integrity of the mitochondrial DNA or on the presence of the drug efflux pump Pdr5. Instead, we show that the increased dosage of Mge1 plays a protective role by retaining increased amounts of ergosterol upon fluconazole treatment. Iron metabolism and, more particularly, Fe-S cluster formation are involved in regulating this process, since the responsible Hsp70 chaperone, Ssq1, is required. Additionally, we show the necessity but, by itself, insufficiency of activating the iron regulon in establishing the Mge1-related effect on drug susceptibility. Finally, we confirm a similar role for Mge1 in fluconazole susceptibility in the pathogenic fungi *Candida glabrata* and *Candida albicans*.

## IMPORTANCE

Although they are mostly neglected compared to bacterial infections, fungal infections pose a serious threat to the human population. While some of them remain relatively harmless, infections that reach the bloodstream often become lethal. Only a few therapies are available, and resistance of the pathogen to these drugs is a frequently encountered problem. It is thus essential that more research is performed on how these pathogens cope with the treatment and cause recurrent infections. Baker’s yeast is often used as a model to study pathogenic fungi. We show here, by using this model, that iron metabolism and the formation of the important iron-sulfur clusters are involved in regulating susceptibility to fluconazole, the most commonly used antifungal drug. We show that the same process likely also occurs in two of the most regularly isolated pathogenic fungi, *Candida glabrata* and *Candida albicans*.

## INTRODUCTION

Fungal infections pose a significant threat to the health of humans and other organisms. Some of these infections are superficial and merely impose a mild form of inconvenience to the patient, while others are invasive, causing severe disease and, potentially, death. Once an invasive infection is established, the likelihood of survival for the patient rarely exceeds 50% (1). The gravity of fungal infections and the concomitant importance of searching for new and better antifungal therapies are generally underappreciated. The number of drugs available against fungal infections is limited, and those that are commonly used often suffer from being fungistatic rather than fungicidal (2, 3). The azoles, with fluconazole (flu) being the most studied, comprise one of these commonly used, fungistatic classes of antifungals (4). The azoles target the ergosterol biosynthesis pathway, more particularly, the lanosterol 14α-demethylase (Erg11). This enzyme is essential in *Saccharomyces cerevisiae*, making the nonfungicidal nature of these drugs paradoxical (5, 6). Resistance to azoles is regularly caused by increased expression of genes encoding efflux pumps, causing overexpression of or altering the target gene by point mutations or generating cellular responses to cope with stress (4). The fungus can, however, also obtain certain transient, metabolic or epigenetic, adaptations that confer decreased susceptibility to the antifungal agent. This slow residual growth at inhibitory concentrations of the drug is called tolerance or trailing growth and hypothetically also generates the time needed for and the possibility of directional selection promoting the acquirement of alterations in the genome, causing resistance (7, 8).

In this project, we isolated *MGE1* as a multicopy suppressor of fluconazole susceptibility in *S. cerevisiae*. Mge1 is a cochaperone for members of the Hsp70 family of chaperones (9, 10), which serve functions in several cellular processes such as protein folding, preventing protein aggregation, protein translocation, targeted degradation, and adjusting the activity of regulatory proteins (11, 12). This cochaperone was discovered as a member of the mitochondrial import system, translocating proteins across the inner membrane into the matrix of the mitochondria (9, 13–15). The Hsp70 molecule involved is Ssc1, which is, like Mge1, an essential protein and is involved in refolding of denatured proteins (9, 16–18). Mge1 also functions as the nucleotide exchange factor of Ssq1, another Hsp70 chaperone, which is involved in the Fe-S cluster biosynthesis pathway (19). Fe-S clusters are essential cofactors involved in redox, catalytic, and regulatory processes, including the regulation of the iron starvation response (20–24). Ssq1 is responsible for transferring the assembled Fe-S cluster from the Isu1 scaffold to the target protein by destabilizing the connection between the cluster and this scaffold (25). In contrast to Ssc1 and Mge1, Ssq1 is not essential because when Ssq1 is depleted, Ssc1 can probably take over part of its function (26). We showed earlier that iron metabolism is involved in regulating susceptibility to fluconazole, since addition of the iron chelator doxycycline to fluconazole-treated *Candida albicans* and *S. cerevisiae* cells reduces or even completely abolishes tolerance (7, 27). In this paper, we provide evidence of the involvement of Fe-S cluster metabolism and signaling through the iron regulon in the Mge1-dependent regulation of fluconazole susceptibility in *S. cerevisiae*. We also demonstrate that this altered susceptibility is accompanied by modulation of the metabolic flux through the ergosterol synthesis pathway. Finally, we show that overexpressing the orthologues of *MGE1* in the pathogenic fungi *C. glabrata* and *C. albicans* affects fluconazole susceptibility in a similar way. As such, elucidating this apparently conserved fungal mechanism may yield interesting new targets for drug development.

## RESULTS

### Increased dosage of Mge1 acts as a suppressor of susceptibility to fluconazole in *S. cerevisiae*.

**FIG 1:**
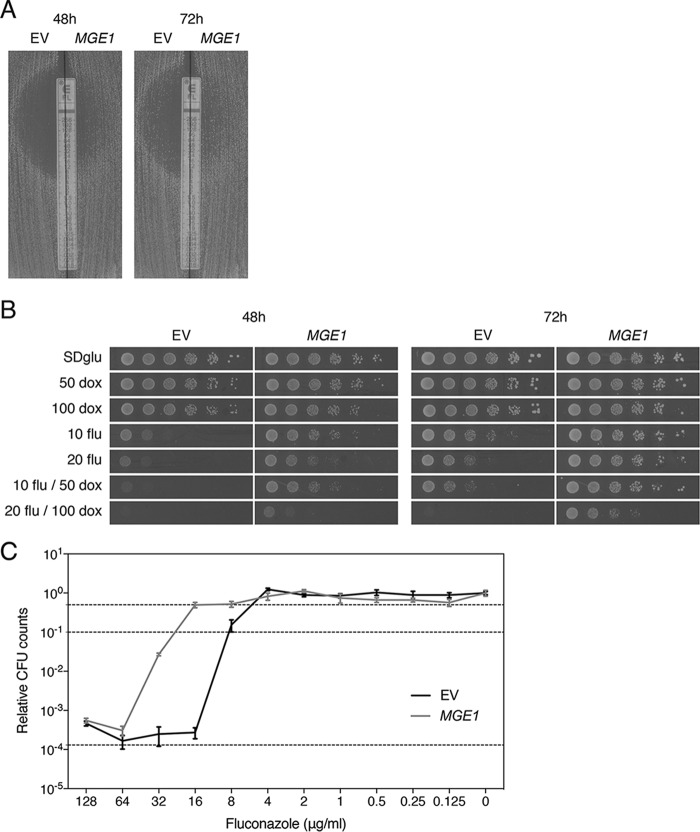
*MGE1* overexpression improves growth of the wild-type *S. cerevisiae* strain on fluconazole. (A) Etest analysis of the overexpression strain (*MGE1*) and control strain (EV). (B) Serial dilutions of both strains were spotted on SDglu medium containing fluconazole (flu; 10 or 20 μg/ml) and/or doxycycline (dox; 50 or 100 μg/ml). Pictures were taken after 48 and 72 h of incubation at 30°C. (C) Tolerance assay. Data represent dose-response curves determined for both strains, with dotted lines indicating 50% (upper line) and 90% (middle line) growth inhibition and the initial inoculum (lower line). No significant difference was observed in trailing growth between the overexpression strain and control strain (*P* = 0.731 for 128 μg/ml flu and *P* = 0.381 for 64 μg/ml flu, tested by two-way ANOVA with Bonferroni correction).

Aiming to identify new regulators of fluconazole susceptibility, we performed a screening of BY4742 transformed with multicopy plasmids, containing parts of the *S. cerevisiae* genomic library obtained from F. Lacroute (28). To reduce the background growth of the reference strain on the screening medium containing supra-minimum inhibitory concentrations (MICs) of fluconazole, we added the iron chelator doxycycline, for which we and others reported a synergistic effect with fluconazole earlier (7, 27). The resulting reduction of background growth allowed us to more clearly distinguish true multicopy suppressors of fluconazole susceptibility. Using these sensitized screening conditions (10 μg/ml fluconazole and 50 μg/ml doxycycline), we isolated the Hsp70 cochaperone Mge1, next to Erg11, as a dosage-dependent suppressor of susceptibility to fluconazole. We subcloned the *MGE1* fragment (containing the promoter, open reading frame [ORF], and terminator) from the pFL44 plasmid into YEPlac195 and verified overexpression in transformants using quantitative reverse transcription-PCR (qRT-PCR), which yielded a fold increase of 11.7 (standard error of the mean [SEM], 1.40) compared to the control strain. This increased expression causes a strong decrease in susceptibility to fluconazole compared to the empty vector control. The improved growth of the transformed BY4742 strain (indicated as *MGE1* in all figures) compared to the control (with empty YEPlac195, indicated as EV) was visualized by means of the Etest method and spot assays (Fig. 1A and B). The MIC_flu_ of these strains was determined by Etest analyses and broth microdilution assays. All experiments were done with at least three biological repeats, showing consistent results. The MIC_flu_ values are depicted in Table 1. We can conclude from these data that overexpression of *MGE1* causes a decrease in the susceptibility to fluconazole in *S. cerevisiae* and that this effect is more clearly visible under sensitized conditions where doxycycline is added to the medium. From the broth microdilution assay, we not only were able to determine the MIC_50_ and MIC_90_ of the mutant compared to the control but also defined the effect of the overexpression on the growth at supra-MICs of fluconazole, called tolerance. Figure 1C shows that, although the MIC_50_ and MIC_90_ change clearly when *MGE1* is overexpressed, there is no significant difference between the colony forming unit (CFU) counts at higher fluconazole concentrations. Therefore, in the following parts of this article, we use only the MIC_flu_ as a readout of drug susceptibility.

**TABLE 1:** The effect of *MGE1* overexpression on the MIC_flu_ of several strains^d^

Strain	MIC_flu_ (μg/ml)		
	Etest	Broth microdilution assay	
		MIC_50_	MIC_90_
*S. cerevisiae* BY4742 EV	6–8	8–16	16
*S. cerevisiae* BY4742 *MGE1*	24–32	16–32	32–64
*S. cerevisiae ira2Δ* EV	4–6	8–16	8–16
*S. cerevisiae ira2Δ MGE1*	12–16	16–32	16–32
*S. cerevisiae yme1Δ* EV	12–16	8–16	16–32
*S. cerevisiae yme1Δ MGE1*	32–48	16–32	32–64
*S. cerevisiae opi1Δ* EV	12–16	8–16	8–16
*S. cerevisiae opi1Δ MGE1*	48–64	32–64	32–64
*S. cerevisiae* rho^0^ EV	24–32	16–32	16–32
*S. cerevisiae* rho^0^ *MGE1*	>256	32–64	32–64
*S. cerevisiae pdr5Δ* EV	0.25	0.5–1	0.5–1
*S. cerevisiae pdr5Δ MGE1*	0.75	0.5–1	1–2
*S. cerevisiae upc2Δ* EV	4–6	4–8	4–8
*S. cerevisiae upc2Δ MGE1*	24–32	8–16	16–32
*S. cerevisiae tom70Δ* EV	6–8	8–16	8–16
*S. cerevisiae tom70Δ MGE1*	16–24	16–32	32–64
*S. cerevisiae ecm10Δ* EV	6–8	8–16	16–32
*S. cerevisiae ecm10Δ MGE1*	24–32	32–64	32–64
*S. cerevisiae ssq1Δ* EV	2–4^a^	2–4^a^	2–4^a^
*S. cerevisiae ssq1Δ MGE1*	1–1.5^a^	—^b^	—^b^
*S. cerevisiae aft1Δ* EV	4–6	8–16	16
*S. cerevisiae aft1Δ MGE1*	4–6	8–16	8–16
*S. cerevisiae aft2Δ* EV	4–6	8–16	8–16
*S. cerevisiae aft2Δ MGE1*	32–48	16–32	32–64
*S. cerevisiae* BY4742	6–8	8–16	16–32
*S. cerevisiae fra1Δ*	6–8	8–16	16–32
*C. glabrata* HTL EV	8 (16–24)^c^	2–4	4–8
*C. glabrata* HTL *pTDH3-CgMGE1*	24 (48–64)^c^	4–8	8–16
*C. glabrata* HTL *pPGK1-CgMGE1*	16 (48–64)^c^	2–4	8–16

a Data were determined after 72 h on SCglu (latter only for Etest).

b —, data could not be determined due to low growth.

c RPMI medium with 0.2% (or 2%) glucose was used.

d Values were determined by Etest and broth microdilution analysis. *MGE1*, *MGE1* overexpression; EV, empty vector control.

Next, we aimed to check the effect of fluconazole on *MGE1* expression under our experimental conditions. We performed qRT-PCR experiments on a wild-type BY4742 strain in the absence or presence of 20 μg/ml fluconazole. The expression of the gene decreased 2-fold in the presence of the drug, indicating that Mge1 itself might be a direct or indirect target of fluconazole {relative expression level with SEM, 1 ± 0.046 versus 0.498 ± 0.045 for 0 versus 20 μg/ml fluconazole with *P* = <0.001 [paired Student’s *t* test on log_2_(Y) transformed data]}.

### Mge1 can induce fluconazole resistance independently of rho^0^ formation and Pdr5.

*S. cerevisiae* cells can lose part or all of their mitochondrial genome, generating so-called rho^−^ or rho^0^ cells, respectively (29, 30). It has been reported that such cells acquire resistance to certain chemicals such as fluconazole, though the underlying mechanisms are not yet fully known (31). Petite-negative strains contain nuclear mutations that render the loss of (part of) the mitochondrial genome lethal (32). Consequently, these strains cannot form rho^0^ or rho^−^ cells. To verify whether decreased fluconazole susceptibility of the *MGE1* overexpression strain might be caused by increased generation of rho^0/−^ cells, we transformed petite-negative strains with the overexpression vector. We chose three mutants involved in seemingly independent processes. The null mutants of *OPI1*, *IRA2*, and *YME1* were all discovered to be dependent on mitochondrial DNA (mtDNA) (33, 34). For these strains, the MIC_flu_ tests were performed in minimal synthetic defined glucose (SDglu) medium as well as rich yeast extract-peptone-dextrose (YPD) medium, as it has been suggested that some petite-negative strains depend only on their mtDNA in rich medium (34). Figure S1A  in the supplemental material and the MIC_flu_ values in Table 1 and in Table S4 in the supplemental material show the sustained effect of *MGE1* overexpression on growth of the petite-negative strains in the presence of fluconazole, arguing against the hypothesis that rho^0/−^ cells are the sole cause of improved growth on fluconazole. We have to take into account, however, that overexpression of *MGE1* could potentially suppress the dependency of the petite-negative mutants on their mtDNA. Nevertheless, overexpression of *MGE1* also causes an increase in the MIC_flu_ of a rho^0^ strain, as can be seen in Table 1 and Fig. S1A, confirming our hypothesis more incontestably. Taken together, these data suggest that the decreased susceptibility to fluconazole in *MGE1*-overexpressing cells is not (solely) caused by increased generation of rho^0/−^ cells.

10.1128/mBio.00201-17.1 FIG S1  Etest analysis of the *S. cerevisiae* strains under study. Pictures were taken after 48 h (or 72 h) of incubation at 30°C. (A) *MGE1* overexpression in different petite-negative strains and a rho^0^ strain, tested on SDglu and YPD media. (B) Deletion of *PDR5* in BY4742 and rho^0^ and *MGE1* overexpression in the *pdr5Δ* strain. (C) *MGE1* overexpression compared to EV control results in a *upc2Δ* strain. (D) *tom70Δ* strain compared to the BY4742 wild type and *MGE1* overexpression in this deletion strain. (E) *ecm10Δ* strain compared to the BY4742 wild type and *MGE1* overexpression in this deletion strain. (F) *ssq1Δ* strain compared to the BY4742 wild type and *MGE1* overexpression in this deletion strain [SC(-URA)glu medium was used here, results seen after 48 and 72 h are shown]. (G) *MGE1* overexpression compared to EV control in *aft1Δ* and *aft2Δ* strains. (H) *fra1Δ* strain compared to the BY4742 wild type. Download FIG S1, PDF file, 8 MB. Copyright © 2017 Demuyser et al. 2017 Demuyser et al. This content is distributed under the terms of the Creative Commons Attribution 4.0 International license.

Overexpression of genes encoding drug efflux pumps is another well-known method of acquiring resistance to drugs that can penetrate the cell. In *Candida* species, expression of genes of the CDR and MDR families, encoding ABC transporters and major facilitators, respectively, are often found upregulated in clinically isolated resistant strains (35–37). The orthologue of the *C. albicans CDR1* gene in *S. cerevisiae* is *PDR5*. It was verified that *PDR5* expression is augmented in rho^0/−^ cells compared to rho^+^ cells. The acquired resistance to several types of chemicals is thought to be caused by this phenomenon (31). Expression levels of *PDR5* were higher in the *MGE1* overexpression strain, indicating that Pdr5 might have been involved in the increased growth on fluconazole {relative expression level ± SEM for 0 μg/ml fluconazole and EV versus *MGE1*, 1.000 ± 0.047 versus 1.596 ± 0.165 with *P* < 0.01; for 20 μg/ml fluconazole and EV versus *MGE1*, 1.482 ± 0.086 versus 2.494 ± 0.088 with *P* < 0.001 [Bonferroni-corrected two-way analysis of variance (ANOVA) of log_2_(Y) transformed data]}. To determine whether this increase was the sole cause of the decreased susceptibility of the *MGE1* overexpression strain, we assessed the effect of *MGE1* overexpression on the MIC_flu_ of the *pdr5Δ* strain. It can be seen from Table S4 and Fig. S1B that deletion of *PDR5* in the rho^0^ background reduced the MIC_flu_ to the same level as deletion of *PDR5* in the wild-type BY4742 background, reinforcing the notion that much of the fluconazole resistance of rho^0/−^ cells is due to upregulation of *PDR5* expression. Overexpressing *MGE1* in a *pdr5Δ* strain still resulted in a significant increase of the MIC_flu_ from 0.25 to 0.75 μg/ml (Table 1; Fig. S1B) indicating that, while increased expression of *PDR5* in cells with an elevated dosage of Mge1 may still play a minor role, Mge1 can induce fluconazole resistance independently of the efflux pump.

### Overexpression of *MGE1* increases the residual amount of ergosterol after treatment with fluconazole independently of the expression of fluconazole-induced *ERG* genes.

**FIG 2:**
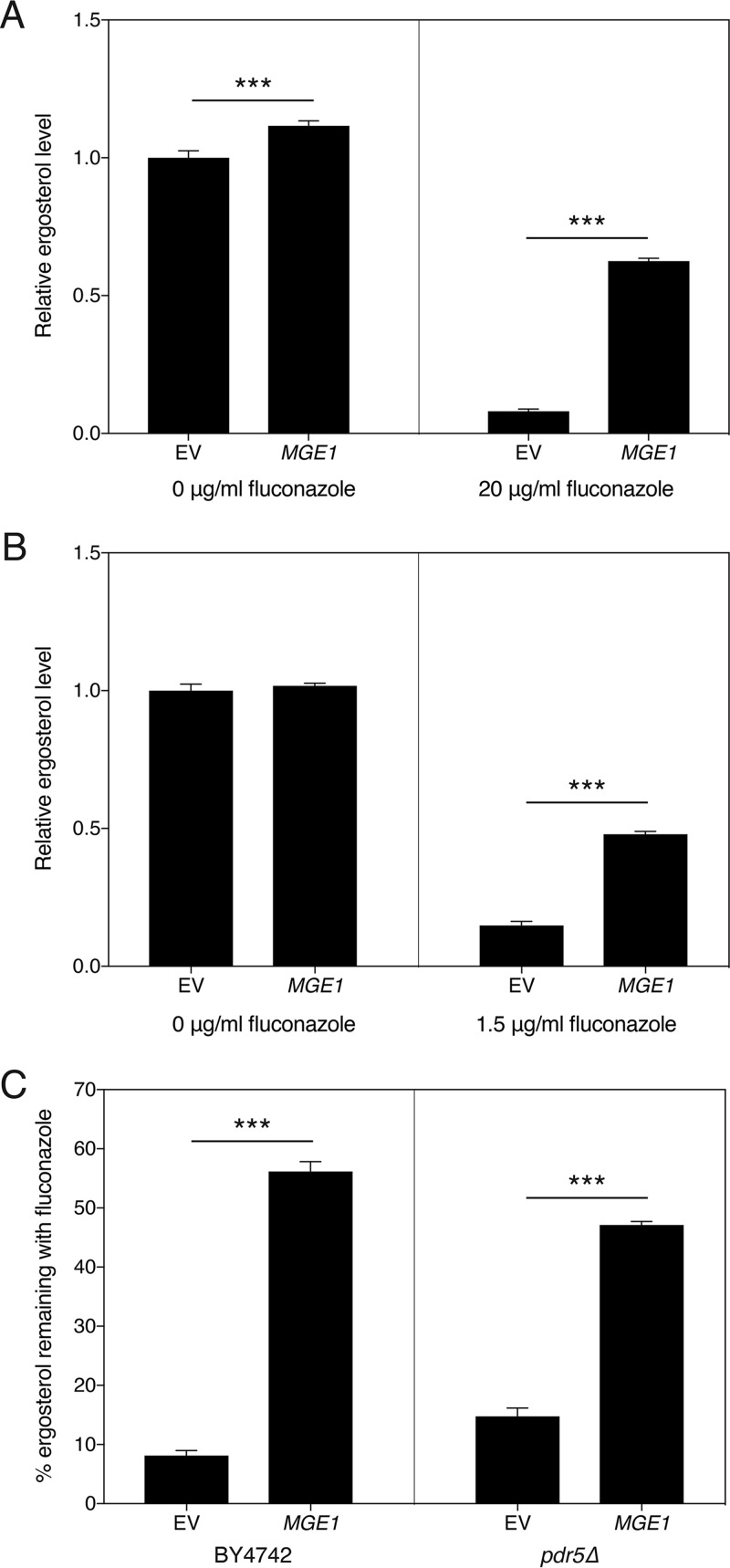
Ergosterol levels are less affected by fluconazole when *MGE1* is overexpressed. *S. cerevisiae* cells were grown in SDglu medium for 24 h, in the presence or absence of fluconazole. (A and B) Ergosterol levels for transformants in the BY4742 background (A) and *pdr5Δ* background (B) are displayed. We note that for the *pdr5Δ* strain, a smaller amount of fluconazole had to be used, due to the increased sensitivity to the drug. The values were calculated relative to the average of the values from the untreated samples. For panels A and B, the interaction between both parameters was statistically significant (*P* < 0.001). (C) Percentage of residual ergosterol for both backgrounds, after fluconazole treatment. Statistical analysis was conducted by two-way ANOVA with Bonferroni correction (A and B) and an unpaired Student’s *t* test (C); ***, *P* < 0.001.

**FIG 3:**
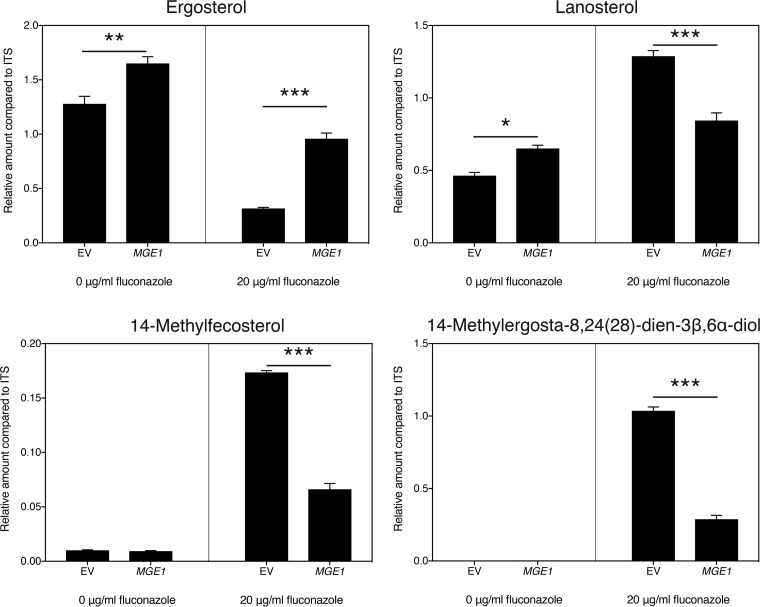
*MGE1* overexpression alters the level of several sterols. Cells were grown in SDglu medium for 24 h in the presence or absence of fluconazole. Sterol levels were determined by GC-MS and are displayed for ergosterol, lanosterol, 14-methylfecosterol, and 14-methylergosta-8,24(28)-dien-3β,6α-diol. The values were calculated relative to the internal standard (ITS; cholestane). The interaction between the two parameters was significant for each sterol (*P* < 0.05). Statistical analysis was conducted by two-way ANOVA with Bonferroni correction; *, *P* < 0.05; **, *P* < 0.01; ***, *P* < 0.001. Data from other sterols that were detected, but that were generally less abundant or could not be identified, are displayed in Fig. S2A.

As already shown by Arthington-Skaggs et al. for *C. albicans* (38), resistance to fluconazole often correlates with higher residual ergosterol levels after drug application. To investigate the possible role of ergosterol in mediating the effect of *MGE1* overexpression on fluconazole susceptibility, we measured ergosterol in the overexpression mutant. As can be seen from Fig. 2A, in the absence of fluconazole, there was only a small difference between the control and the strain overexpressing *MGE1*. Upon treatment with fluconazole, however, the fraction of ergosterol remaining in the mutant was significantly higher than the control (Fig. 2A and C). This phenotype was again independent of Pdr5, since the effect was still visible in the *pdr5Δ* mutant (Fig. 2B and C). To elucidate how *MGE1* overexpression affects sterol synthesis in general, we performed gas chromatography-mass spectrometry (GC-MS) analysis of the sterols isolated from our strains, in the absence and presence of fluconazole (Fig. S2A and B). As fluconazole targets Erg11, lanosterol accumulates and ergosterol levels decrease in the presence of the drug. Under these conditions, lanosterol is also converted to 14-methylfecosterol and ultimately to the toxic compound 14-methylergosta-8,24(28)-dien-3β,6α-diol, which represents an important aspect of the mode of action of the drug (39, 40). Interestingly, we saw that, compared to the control strain, overexpression of *MGE1* reduced the metabolic flux that leads to toxic sterol formation, thereby maintaining a higher flux toward ergosterol production (Fig. 3).

10.1128/mBio.00201-17.2 FIG S2  Sterols detected by GC-MS in the *MGE1* overexpression strain compared to the control. (A) Strains were grown in SDglu medium for 24 h, in the absence or presence of fluconazole. The sterol composition of EV and *MGE1* strains was analyzed under both sets of conditions. The abundance of each compound was calculated as the peak area relative to cholestane (internal standard) based on CG-MS analysis of four biological replicates. RT, retention time; RRT, relative retention time. (B) Schematic representation of the main ergosterol biosynthesis pathway (adapted from reference 78), with the sterols that we identified in red. The symbol depicted next to the identified sterol indicates how the fluconazole-induced change in this sterol is altered upon overexpression of *MGE1* (only significant changes are shown [tested by two-way ANOVA with Bonferroni correction]). Download FIG S2, PDF file, 0.8 MB. Copyright © 2017 Demuyser et al. 2017 Demuyser et al. This content is distributed under the terms of the Creative Commons Attribution 4.0 International license.

As Erg11 is the target of fluconazole and the point in the sterol synthesis pathway where progress to either ergosterol or the toxic sterol is defined, it seems valid to hypothesize that Erg11 might be the enzyme linking Mge1 to ergosterol. We checked the expression levels and protein levels of *ERG11* and the Erg11 protein, respectively, in the mutant and control strains in both the absence and presence of fluconazole. Remarkably, gene expression levels and protein levels remained the same and were reduced, respectively, rather than upregulated in the *MGE1* overexpression strain (Fig. S3A and B). For the Western blot analysis, we used an anti-hemagglutinin (anti-HA) antibody and the AFc202 strain, where *ERG11* was tagged chromosomally with a 3× HA tag, thus representing native expression. We verified that the MIC_flu_ of this mutant is similar to that of the BY4742 wild-type strain, as can be seen in Table S4. Apart from *ERG11*, other genes encoding ergosterol biosynthesis enzymes have also been shown to be induced upon azole treatment (41–44). Still, we found that overexpression of *MGE1* did not significantly upregulate the expression of *ERG2*, *ERG3*, *ERG4*, *ERG5*, *ERG6*, *ERG7*, *ERG8*, *ERG9*, *ERG12*, *ERG19*, *ERG24*, or *ERG25* under either control or fluconazole-treated conditions (Fig. S3A). As described by MacPherson et al. in 2005 for *C. albicans* (43), Upc2 confers resistance to antifungals by modulating expression of certain genes involved in ergosterol biosynthesis. We confirm here that Mge1 did not function upstream of Upc2 in increasing the MIC_flu_, since *MGE1* overexpression still caused a decrease in fluconazole susceptibility in an *upc2Δ* strain (Table 1; Fig. S1C). Additionally, Upc2 did not influence *MGE1* expression, as can be seen from Fig. S3C. In summary, although Mge1 alters the flux through the sterol synthesis pathway, thereby maintaining increased ergosterol levels and decreasing toxic sterol levels, this does not appear to be mediated by changing the expression level of the fluconazole-dependent genes encoding the main biosynthesis enzymes in this sterol pathway.

10.1128/mBio.00201-17.3 FIG S3  Increased dosage of Mge1 does not cause Erg11 levels or *ERG*-related gene expression to increase. (A) Expression of none of the *ERG* genes increased significantly when *MGE1* was overexpressed. Cells were grown for 24 h in SDglu medium, in the presence or absence of 20 μg/ml fluconazole. The results of qRT-PCR analysis are displayed as the average of log_2_(Y) transformed values with the SEM. The values were calculated relative to the average of the values from the untreated samples. Statistical analysis was conducted on the transformed values by two-way ANOVA with Bonferroni correction; *, *P* < 0.05; **, *P* < 0.01. (B) Erg11 protein levels decrease slightly when *MGE1* is overexpressed. Cells from strain AFc202 were grown for 24 h in SDglu medium, in the presence or absence of 20 μg/ml fluconazole. The blots were probed with anti-HA or anti-Pgk1 antibodies (loading control). (C) Expression of *MGE1* does not alter significantly in an *upc2 Δ* strain compared to the wild type. The results of qRT-PCR analysis are displayed as the average of log_2_(Y) transformed values with the SEM relative to BY4742 values. Statistical analysis was conducted on the transformed values by unpaired Student’s *t* test. Download FIG S3, PDF file, 0.3 MB. Copyright © 2017 Demuyser et al. 2017 Demuyser et al. This content is distributed under the terms of the Creative Commons Attribution 4.0 International license.

### The Mge1-dependent decrease in fluconazole susceptibility requires the mitochondrial chaperone Ssq1.

**FIG 4:**
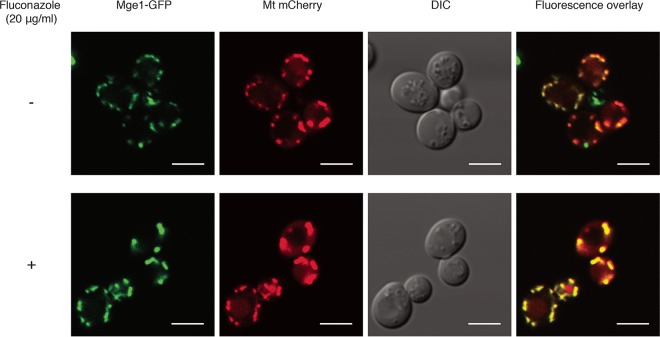
Mge1 localizes to the mitochondria. The BY4742 strain expressing both *MGE1-GFP* and mitochondrially targeted (Mt) *mCherry* was incubated for 24 h in the absence or presence of 20 μg/ml fluconazole, pictures were taken afterward. The scale bar represents 5 μm. DIC, differential interference contrast.

Both known processes involving Mge1, i.e., Fe-S cluster formation and protein import across the inner mitochondrial membrane, are localized to the mitochondria. Although the literature also reports on the localization of Mge1 to this organelle (45), the experimental procedures used always consisted of *in vitro* rather than *in vivo* methods. To verify that Mge1 indeed functions inside the mitochondria, in the absence as well as the presence of fluconazole, we checked its localization by fluorescence microscopy. From Fig. 4, it can be seen that Mge1 localized to the mitochondria in the overexpression mutant, under both conditions. This indicates that the function by which Mge1 causes a decrease in the susceptibility to fluconazole must also be confined to this organelle. It was verified that *MGE1*-*GFP* overexpression still caused an increased MIC_flu_ level (Table S4).

To further elucidate the mode of action by which Mge1 converts resistance to fluconazole, we postulated that this phenotype is effectuated by either of the downstream Hsp70 proteins. Ssc1 is part of the TIM23 complex spanning the inner mitochondrial membrane and works as an ATPase, providing energy to transport proteins into the mitochondria. A paralog of Ssc1, Ecm10, probably arose through genome duplication (82% amino acid identity) and is thought to have functions that overlap those of Ssc1 (46–48). It has been shown that Ecm10 also interacts with Mge1 (48). Ssq1 shows limited homology with Ssc1 (52% amino acid identity) and plays a role in one of the initial steps of Fe-S cluster formation, together with Mge1 (19). To determine whether the effect of *MGE1* overexpression on fluconazole susceptibility operates through Ssc1, Ecm10, or Ssq1, we verified if the resistance phenotype is still observed in mutants with a defect in either of the downstream pathways. Tom70 is part of the translocase of the outer mitochondrial membrane (TOM) complex, playing a role in recognizing and importing mitochondrial proteins (49, 50). Deletion of the *TOM70* gene affects protein import into the mitochondria (50). The MIC_flu_ of the *tom70Δ* strain was equal to that of the wild type (Fig. S1D; Table S4), and overexpression of *MGE1* in this strain resulted in an increase in the MIC_flu_ similar to that seen with the wild type, indicating that full protein import into the mitochondria is not essential for the Mge1-related effect on fluconazole susceptibility (Fig. S1D; Table 1). As mentioned before, Ecm10 is a paralog of Ssc1. Since deletion of *ECM10*, in contrast to *SSC1*, is viable, we decided to see if overexpression of *MGE1* in this strain would still cause an increase in the MIC_flu_. As can be seen from Fig. S1E and Table S4, the *ecm10Δ* strain had an MIC_flu_ similar to that of the wild-type BY4742 strain. Overexpression of *MGE1* in this strain changed this MIC_flu_ in the same way as was seen with BY4742 (Fig. S1E; Table 1), implying that Ecm10 is also not involved. Deletion of *SSQ1* is viable; therefore, we also tested the MIC_flu_ of the *ssq1Δ* strain and found it to be significantly lower than that of the BY4742 wild-type strain (Fig. S1F; Table S4), in agreement with a previous report by Dagley et al. (51). Overexpression of *MGE1* in the *ssq1Δ* strain yielded remarkably few and slow-growing transformants (our unpublished observations), suggesting that combining a deletion of *SSQ1* with overexpression of *MGE1* alters the cells’ fitness. Additionally, when *MGE1* was overexpressed, the MIC_flu_ of the *ssq1Δ* strain did not increase compared to that of the empty vector control and even displayed a decrease (Fig. S1F; Table 1). This suggests that Ssq1 is necessary to establish the Mge1-mediated effect on fluconazole susceptibility.

### Activation of the iron regulon is necessary but not sufficient for Mge1 to exert its effect on fluconazole susceptibility.

**FIG 5:**
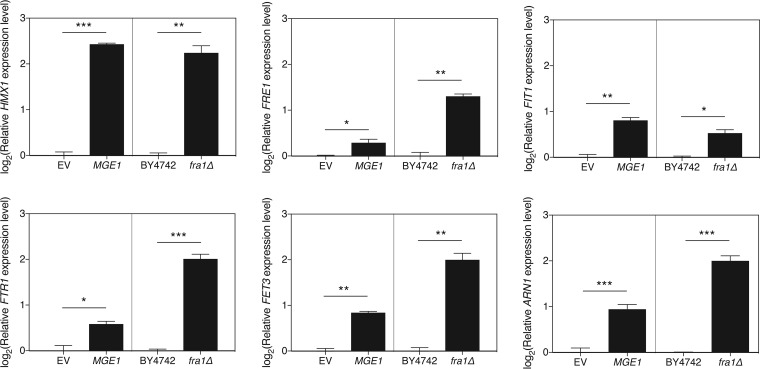
Expression of typical iron regulon genes increases upon overexpression of *MGE1* or deletion of *FRA1*. Expression of the representative iron regulon genes *HMX1*, *FRE1*, *FIT1*, *FTR1*, *FET3*, and *ARN1* was analyzed by qRT-PCR. For each gene, the left panel shows the effect of overexpressing *MGE1* in the BY4742 strain versus the EV control. The right panel shows comparisons of the levels of gene expression between BY4742 and *fra1Δ* strains. Results are displayed as the average of log_2_(Y) transformed values with the SEM. The values were calculated relative to the averages of the values from the respective controls. Statistical analysis was conducted by unpaired Student’s *t* test with Bonferroni correction; *, *P* < 0.05; **, *P* < 0.01; ***, *P* < 0.001.

It seems evident, from the literature and previous findings described above, that iron plays a role in regulating susceptibility to fluconazole (7, 27). In an attempt to clarify how this happens and how Mge1 provides a link in this process, we investigated the possible involvement of the iron regulon. Aft1 is a transcriptional regulator which induces transcription of genes involved in the recovery of iron upon iron starvation (22, 23). *AFT2* encodes a paralog of *AFT1*, which arose through gene duplication. The proteins encoded by the two genes have partially overlapping functions, with Aft2 being responsible for the iron metabolism when Aft1 is not present (24). Overexpression of *MGE1* still reduced the susceptibility to fluconazole in an *aft2Δ* strain, but this effect was lost in the *aft1Δ* strain (Fig. S1G; Table 1). As Aft1 is necessary for the Mge1-related effect, we speculated that, upon overexpression of the cochaperone gene, the expression of iron regulon genes might also be induced. We confirmed this for six iron regulon genes (Fig. 5). Next, we questioned whether mere activation of the iron regulon could explain the Mge1-regulated effect on fluconazole susceptibility or whether this is only part of the mechanism. Fra1 is a negative regulator of the iron regulon. In the presence of an as-yet-unknown signal coming from the Fe-S cluster metabolism in the mitochondria, Fra1 forms a complex with Fra2, Grx3, and Grx4 and inhibits the translocation of Aft1 to the nucleus, thereby inhibiting transcription of the iron regulon genes (52). Deletion of *FRA1* was shown to induce the iron regulon, even in the presence of large amounts of iron (52). We confirmed that deletion of *FRA1* induced expression of the iron regulon genes in our experimental setup as well. In Fig. 5, we show that the induction of expression in the *fra1Δ* strain was always similar to or higher than the induction seen upon *MGE1* expression, indicating the validity of the comparison. If activation of the iron regulon were the sole mechanism by which *MGE1* overexpression leads to fluconazole resistance, the *fra1Δ* strain should also show an increased MIC_flu_ compared to that of the wild-type BY4742 strain. However, Table 1 and Fig. S1H show that this is not the case, indicating the requirement of yet another unknown process to work in conjunction with iron regulon activation in establishing fluconazole resistance downstream of Mge1. Thus, activation of the iron regulon is necessary but is insufficient by itself to induce resistance against fluconazole downstream of Mge1.

### Increased dosage of Mge1 also acts as a suppressor of susceptibility to fluconazole in *C. glabrata*.

*C. glabrata* and *C. albicans* are two of the most frequently isolated pathogenic fungi in humans (53). For the past few years, *C. glabrata* infections have been on the rise in northern Europe and the United States, indicating a need for specific research and drug development (54). Its evolutionarily close relationship with *S. cerevisiae* (55) implies that the phenotype that we observed for *S. cerevisiae MGE1* (*ScMGE1*) overexpression with respect to susceptibility to fluconazole might also apply to *MGE1* in *C. glabrata*. The closest *C. glabrata* orthologue of *S. cerevisiae* Mge1 is encoded by CAGL0J03850g, which is indicated as an uncharacterized ORF in the Candida Genome Database (CGD) (56). Comparing the protein sequence of *S. cerevisiae* Mge1 to that of its orthologue in *C. glabrata* yielded an amino acid identity of 68%. We thus refer to the *C. glabrata* orthologue as *C. glabrata* Mge1 (*Cg*Mge1). To investigate the effect of *CgMGE1* overexpression on susceptibility to fluconazole, we created two plasmids expressing the *CgMGE1* ORF, together with its terminator, from either the *CgPGK1* promoter or the *CgTDH3* promoter. Overexpression of *MGE1* in the transformed 2001HTL strains was verified using qRT-PCR, yielding fold increases of 24.9 (SEM, 5.54) for the *CgPGK1* promoter and 42.9 (SEM, 4.0) for the *CgTDH3* promoter compared to the control. Both Etest and broth microdilution analyses indicated that overexpression of *CgMGE1* in *C. glabrata* also increased the MIC_flu_ (Table 1; Fig. S4A). The microdilution assay was performed on RPMI medium containing 0.2% glucose, while the Etest analysis was performed on RPMI agar plates containing both 0.2% and 2% glucose. The addition of extra glucose generally enhances the ability to visually inspect the MIC_flu_, as formerly shown for *C. albicans* (57). As with *S. cerevisiae*, no significant effect of *CgMGE1* overexpression on tolerance was observed (Fig. S4B). In summary, these data suggest that, similarly to the situation in *S. cerevisiae*, *Cg*Mge1 plays a role in regulating susceptibility to fluconazole in *C. glabrata*.

10.1128/mBio.00201-17.4 FIG S4  Etest analysis and microdilution assay of *CgMGE1* overexpression in the *C. glabrata* 2001HTL strain. (A) Etest analysis, performed on media containing 0.2% or 2% glucose. Pictures were taken after 48 h of incubation at 37°C. (B) Dose-response curves of all strains, with dotted lines indicating 50% (upper line) and 90% (middle line) growth inhibition and the initial inoculum (lower line). *P* = 0.807 (128 μg/ml flu, EV versus p*PGK1*-*CgMGE1*), *P* = 0.857 (64 μg/ml flu, EV versus p*PGK1*-*CgMGE1*), *P* = >0.999 (128 μg/ml flu, EV versus p*TDH3*-*CgMGE1*), and *P* = 0.118 (64 μg/ml flu, EV versus p*TDH3*-*CgMGE1*). *P* values were calculated by two-way ANOVA with Bonferroni correction. Download FIG S4, PDF file, 1.6 MB. Copyright © 2017 Demuyser et al. 2017 Demuyser et al. This content is distributed under the terms of the Creative Commons Attribution 4.0 International license.

### Overexpression of *MGE1* affects both resistance and tolerance in *C. albicans*.

**FIG 6:**
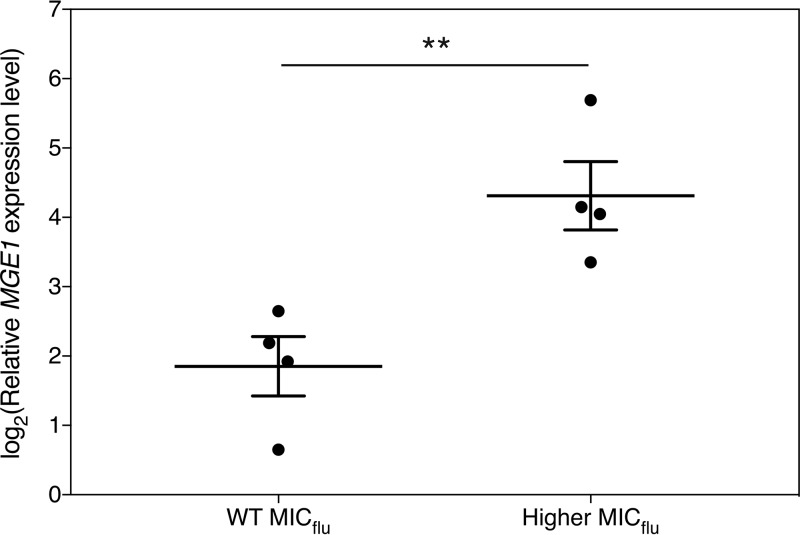
Overexpression level of *CaMGE1* in SC5314 correlates with the MIC_flu_. *C. albicans* strain SC5314 was transformed with plasmid pLDa01 (CIp10-*CaMGE1*), and fluconazole sensitivity was determined with the Etest method. Transformants with MIC_flu_ values that were similar to or higher than those seen with the EV control strains were obtained. For 4 transformants of each group, *CaMGE1* expression was determined by qRT-PCR, and the values were calculated relative to the average of the values from the EV control samples (see Fig. S5). The results are displayed as the average of log_2_(y) transformed values with the SEM along with the separate data points. The statistical analysis was conducted by unpaired Student’s *t* test; **, *P* < 0.01. WT, wild type.

Although the incidence of *C. glabrata* infections is increasing steadily in certain parts of the world, *C. albicans* is still the most prevalent cause of *Candida* infections worldwide (53). The evolutionary distance between this important pathogen and *S. cerevisiae* is, however, bigger than is the case for *C. glabrata*, indicating that *S. cerevisiae* might not be as good a model system for *C. albicans* as it is for *C. glabrata* (55). To check whether Mge1 is also involved in fluconazole susceptibility in *C. albicans*, we generated a plasmid where *C. albicans MGE1* (*CaMGE1*) is under the control of the strong, constitutive *CaACT1* promoter. The CIp10 plasmid integrates in the genome at the *RP10* locus, where it should stably overexpress *CaMGE1* (58). The SC5314 strain was transformed with either the overexpression construct or the empty plasmid as a control. While the control transformants displayed a uniform MIC_flu_ phenotype, overexpression of *CaMGE1* yielded two phenotypes. One group of transformants did not show an alteration in the MIC_flu_ compared to the EV controls, while others showed an increased MIC_flu_ which was mainly visible after 24 h of incubation (Fig. S5A and B). We reasoned that this could have been due to different levels of overexpression of *CaMGE1*, as we also had observed various levels of (over)expression in the past upon transformation of *C. albicans* (our unpublished observations). It is speculated that this might be due to the high plasticity of the *C. albicans* genome (59). Here, we confirm a highly variable level of *CaMGE1* overexpression in our transformants and demonstrate that the observed variation is largely due to the various results with respect to copy number integration of the plasmid in the genome (Fig. S5A). As can be seen in Fig. 6, the highest *CaMGE1* expression levels of the transformants correlated with an increase in the MIC_flu_, indicating that, above a certain threshold of *CaMGE1* expression, increased resistance to fluconazole was detected. Intriguingly, for those strains, we found a decrease in tolerance (Fig. S5C). Our results thus indicate that upon (sufficient) overexpression of *MGE1*, resistance of *C. albicans* to fluconazole is increased, similarly to the situation in *S. cerevisiae* and *C. glabrata*. In contrast to the latter organisms, however, this increased resistance in *C. albicans* seems to come at the cost of a lower tolerance to the same drug.

10.1128/mBio.00201-17.5 FIG S5  Etest analysis and microdilution assay of *CaMGE1* overexpression in the *C. albicans* SC5314 strain. (A) Eight transformants overexpressing *CaMGE1* (LDa01 through LDa08) were analyzed for copy number integration, relative *CaMGE1* expression levels, and MIC_flu_ category compared to the control strain (LDa09). (B) Etest analysis. Pictures were taken after 24 h of incubation at 37°C. Transformants LDa05 and LDa07 are examples of those with an increase in MIC_flu_ and LDa04 and LDa06 as examples of those with an MIC_flu_ similar to that of the controls (LDa09). (C) Tolerance assay assessing trailing growth of 3 transformants showing an increased MIC_flu_ compared to the average of the values (with SEM) determined for three control strains (LDa09, *n* = 3). Due to the variance in *CaMGE1* overexpression (see panel A), CFU counts of the overexpression transformants are shown individually as well as via the average and SEM of the three strains together. Statistical analysis was conducted by two-way ANOVA with Bonferroni correction. ***, *P* = <0.001. Dotted lines indicate 50% (upper line) and 90% (middle line) growth inhibition and the initial inoculum (lower line). Download FIG S5, PDF file, 1.4 MB. Copyright © 2017 Demuyser et al. 2017 Demuyser et al. This content is distributed under the terms of the Creative Commons Attribution 4.0 International license.

## DISCUSSION

In this study, we identified Mge1, a cochaperone involved in Fe-S cluster metabolism and protein import into the mitochondria, as a multicopy suppressor of fluconazole susceptibility (16, 17, 19). When an *S. cerevisiae* mutant strain grows in the presence of an otherwise inhibitory chemical, it is important to consider increased rho^0/−^ formation and drug efflux as possible modes of action (60). Several groups have already reported on a relation between Mge1, or its downstream chaperones, and mtDNA stability (18, 30, 61, 62). However, we show here that neither loss of the mitochondrial DNA nor drug efflux through Pdr5 can solely account for the increased growth of the *MGE1* overexpression strain on fluconazole (33, 34).

The target of fluconazole is Erg11, an enzyme involved in the biosynthesis of ergosterol (5, 6). In this report, we show that ergosterol levels are elevated in an *S. cerevisiae* strain where *MGE1* is overexpressed, a phenomenon which is much more prominent after the addition of fluconazole. It thus seems that Mge1 evokes a protective mechanism by which the cell can retain higher levels of ergosterol upon treatment with fluconazole. A detailed analysis of the sterol spectra of our strains indicated that, upon fluconazole addition, *MGE1* overexpression reduces the metabolic flux toward potential toxic sterols, most notably 14-methylergosta-8,24(28)-dien-3β,6α-diol (39, 40). The reduced accumulation of this sterol, together with the retention of more ergosterol upon fluconazole treatment, illustrates how Mge1 reduces susceptibility to the drug. As Erg11 functions at the cross-section between the pathways leading to either ergosterol production or toxic sterol accumulation, we analyzed the abundance of this enzyme but found no increase at the level of either gene expression or protein abundance upon *MGE1* overexpression. Analysis of the expression of other *ERG* genes, known to be regulated by fluconazole, could also not identify a transcriptional mechanism explaining the observed sterol profiles. It is possible that *MGE1* overexpression specifically alters the enzyme activity of Erg11 or of other ergosterol biosynthesis enzymes, rather than their expression. It remains unclear how Mge1, operating in the mitochondria, would impact ergosterol biosynthesis, which mainly takes place in the endoplasmic reticulum (ER) (63).

To further elucidate how Mge1 function might be linked to fluconazole susceptibility, we looked at the known Mge1 effectors. We demonstrated the involvement of the Hsp70 chaperone Ssq1, as this chaperone is necessary for the cell to retain its MIC_flu_ at the wild-type level and as overexpression of *MGE1* in the *ssq1Δ* strain could not increase fluconazole resistance. Ssq1 is essential for mitochondrial Fe-S cluster metabolism, which somehow functions as an iron-sensing system in the cell, since in the presence of sufficient iron, an inhibitory signal originates from this metabolism and impairs transcription of the iron regulon genes (64). Intriguingly, we found that overexpressing *MGE1* in the wild-type strain causes a significant increase in expression of characteristic iron regulon genes. Furthermore, deleting *AFT1*, the gene encoding the main transcriptional regulator of the iron regulon, impairs the effect of Mge1 on fluconazole susceptibility, indicating the strict dependence of our phenotype on this regulon. It is possible that overloading the cell with Mge1 might impair, rather than increase, the cochaperone’s function. This would then lead to a reduced Fe-S signal, thereby activating the iron regulon and generating fluconazole resistance by modulating sterol synthesis, as shown before under iron-limiting conditions in yeast (65, 66). Several elements argue against such a straightforward mechanism, however. First of all, Schmidt et al. reported that overexpression of *MGE1* increases the activity of Ssq1 (67), implying increased rather than impaired Fe-S cluster biogenesis. Second, impairing Ssq1 function does not lead to fluconazole resistance, as we observed that the *ssq1Δ* strain was more sensitive, and not resistant, to fluconazole. Finally, although we demonstrate the dependency of fluconazole resistance on the activation of the iron regulon, we also clearly show that this is not sufficient, since mere activation of the iron regulon through *FRA1* deletion does not cause any change in the MIC_flu_. It thus remains to be investigated how Mge1 activity is linked to the iron regulon on one side and to fluconazole susceptibility on the other side. It is tempting to speculate that increasing Mge1 activity alters the balance in Fe-S cluster proteins in a specific way, causing fluconazole resistance via two separate pathways: by activating the iron regulon and simultaneously by some other, yet-to-be-elucidated mechanism. Future in-depth analysis of the changes in the Fe-S cluster metabolism upon *MGE1* overexpression would thus represent a valuable system to elucidate this mechanism. This analysis could pinpoint Fe-S species which regulate the resistance to fluconazole through modulation of ergosterol metabolism, i.e., by reducing toxic sterol production and increasing ergosterol retention. At the same time, as the identities of the specific Fe-S species which are involved in regulating the iron regulon are still unknown at present, such an analysis would also provide crucial information on this topic.

Apart from the observations made in *S. cerevisiae*, we also validated the Mge1-related effect on fluconazole resistance in the fungal pathogens *C. glabrata* and *C. albicans*. Very little is known about Fe-S cluster metabolism in either pathogen. The *C. glabrata* orthologue of *ScSSQ1* is uncharacterized (56). The *C. albicans* orthologue was characterized recently (68); these researchers confirmed a role for *Ca*Ssq1 in iron metabolism and iron regulon modulation. More research is necessary to uncover the exact role of the Mge1-Ssq1 module in regulating the susceptibility of fungal cells to fluconazole. Knowledge of this mechanism could provide novel drug targets which would increase the antifungal potential of azoles in combinatorial therapies.

## MATERIALS AND METHODS

### Strains and plasmids.

All *S. cerevisiae* strains used in this study are isogenic with respect to the BY4742 laboratory strain and are listed in Table S1 in the supplemental material. Strain AFc202, carrying a chromosomal 3×HA C-terminal tag at *ERG11*, was constructed by transforming BY4742 with a PCR fragment obtained using primers listed in Table S2 and plasmid pMPY-3xHA as a template (69). Transformants were allowed to pop out the *URA3* marker by homologous recombination, and uracil auxotrophs were selected using 5-fluoroorotic acid (5-FOA). The BY4742 strain was made rho^0^ by repeated growth in the presence of 25 μg/ml ethidium bromide in minimal medium, as described in reference 70. Deletion of *PDR5* in BY4742 and rho^0^ strains was accomplished by amplification of the hygromycin resistance marker gene from plasmid pFA6a-*hphNT1* (71) and consequent transformation. *C. glabrata* and *C. albicans* strains used in the experiments are also listed in Table S1. *C. albicans* strains LDa01 to LDa08 were generated by transforming the StuI-linearized pLDa01 plasmid in SC5314. The LDa09 strain was created similarly by integration of the empty CIp10-*NAT1* plasmid. All specific genotypes were checked by diagnostic PCR.

10.1128/mBio.00201-17.6 TABLE S1  Strains used in this study. Download TABLE S1, PDF file, 0.1 MB. Copyright © 2017 Demuyser et al. 2017 Demuyser et al. This content is distributed under the terms of the Creative Commons Attribution 4.0 International license.

10.1128/mBio.00201-17.7 TABLE S2  Primers used in this study. Download TABLE S2, PDF file, 0.1 MB. Copyright © 2017 Demuyser et al. 2017 Demuyser et al. This content is distributed under the terms of the Creative Commons Attribution 4.0 International license.

Plasmids are listed in Table S3. Plasmid pAFc86 contains *MGE1* under the control of its promoter and terminator. The SnaBI-XhoI fragment from the Lacroute library plasmid was cloned into YEplac195 linearized using SmaI. Plasmid pESc01 is similar to pAFc86, with fusion of *MGE1* to the gene encoding green fluorescent protein [GFP(S65T)]. This plasmid was created by amplification of the *MGE1* promoter, the *MGE1* ORF, the *GFP* gene, and the *MGE1* terminator and assembly of them in the YEPlac195 plasmid using In-Fusion cloning (Clontech). The mitochondria were marked by transforming a plasmid containing *mCherry* fused to a mitochondrial targeting sequence in the appropriate strains (72). Plasmids pESg01 and pESg02 were generated by assembling the *CgPGK1* promoter or *CgTDH3* promoter and the *CgMGE1* ORF and its terminator in the pCgACH backbone (73) by In-Fusion cloning. Plasmid CIp10-*NAT1* was generated by exchanging the *CaURA3* marker together with its promoter and terminator from the CIp10 plasmid (58) for the dominant *C. albicans* optimized *NAT1* gene together with a *CaACT1* promoter and terminator (74), using NotI and SpeI. Another *CaACT1* promoter and terminator were added in the multiple cloning site, opened with MluI-NheI and XhoI-KpnI, respectively. Plasmid pLDa01 was generated by integrating the *CaMGE1* gene in the PstI-ClaI-cut CIp10-*NAT1* vector.

10.1128/mBio.00201-17.8 TABLE S3  Plasmids used in this study. Download TABLE S3, PDF file, 0.1 MB. Copyright © 2017 Demuyser et al. 2017 Demuyser et al. This content is distributed under the terms of the Creative Commons Attribution 4.0 International license.

10.1128/mBio.00201-17.9 TABLE S4  MIC_flu_ values of several strains. Data indicated with a superscript "a" represent the results of overexpression of *MGE1-GFP*. Data indicated with a superscript "b" were determined after 72 h on SCglu medium (the latter was used only for the Etest; BY4742 had MIC_flu_ values of 8 to 12 on this medium). Download TABLE S4, PDF file, 0.1 MB. Copyright © 2017 Demuyser et al. 2017 Demuyser et al. This content is distributed under the terms of the Creative Commons Attribution 4.0 International license.

### Growth conditions: media and chemicals.

*S. cerevisiae* strains were grown in SDglu, unless stated otherwise. This medium contains 0.17% Difco yeast nitrogen base without amino acids or ammonium sulfate, 0.5% ammonium sulfate, and 2% glucose. Liquid medium was pH adapted to pH 5.5. For solid medium, the pH was set at 6.5 and 1.6% agar was added. Depending on the strain, additional amino acids or nucleotides were added according to the method described in reference 75. For spot assays, fluconazole (F8929; Sigma) and doxycycline (D9891; Sigma) were added to the medium at concentrations of 10 or 20 μg/ml and 50 or 100 μg/ml, respectively. The procedure used to screen for multicopy suppressors of susceptibility to fluconazole-doxycycline was described in reference 7. For some specific experiments, YPD medium (containing 1% yeast extract, 2% peptone, and 2% glucose) was used. *C. albicans* and *C. glabrata* strains were pregrown in synthetic complete glucose (SCglu) medium or SC(-HIS)glu medium, composed of SDglu with the addition of complete or drop-out CSM (MP Biomedicals). Assays were carried out in filter-sterilized RPMI 1640 medium with l-glutamine (R6504; Sigma) and buffered with 0.165 M morpholinepropanesulfonic acid at pH 7. Depending on the assay, autoclave-sterilized and precooled agar and/or 1.8% glucose was added to the medium. Cell cultures containing fluconazole or doxycycline were always kept in the dark.

### Determination of fluconazole susceptibility: MIC_flu_ evaluation, tolerance assays, and spot assays.

To determine the MIC_flu_ for the strains, two methods were always used in parallel. In the Etest method (BioMérieux), the MIC_flu_ was determined as the concentration of fluconazole where the halo of growth inhibition/retardation intersected with the strip. Overnight cultures were adjusted to an optical density at 600 nm (OD_600_) of 0.5 in water for *S. cerevisiae* and an OD_600_ of 0.2 for *C. glabrata* and *C. albicans* and were spread on SDglu or RPMI (with 0.2% or 2% glucose) plates. The strips were placed onto the lawn of cells, and the plates were incubated at 30°C or 37°C for 48 h. Broth microdilution assays were conducted according to the Clinical Laboratory and Standards Institute (CLSI) standard methods (76). Round-bottom, UV-sterilized 96-well microtiter plates were used, where all wells were filled with 0.5 to 2.5 × 10^3^ cells/ml, 0 to 128 μg/ml fluconazole in 1/2 dilutions, and SDglu or RPMI medium (the latter with 0.2% glucose). For *C. albicans*, the fluconazole dilution series was set between 0 and 32 μg/ml fluconazole. After incubation of the plates at 30°C or 37°C under nonshaking conditions for 48 h, we measured the OD_600_ of the resuspended cultures in each well to obtain quantitative and objective data. A dose-response curve was created, and the MIC values were calculated by subtracting the background OD values determined for the medium from all measured data points and subsequent normalization to the condition without fluconazole. The concentrations of the drug, between which the relative OD falls below 50 or 10% of the no-drug OD are called the MIC_50_ and the MIC_90_, respectively. To evaluate the drug tolerance of our strains, we generated dose-response curves based on CFU counts for the *MGE1* overexpression strain and empty vector control. The wells of the broth microdilution assay plate were resuspended, and each culture was diluted and plated. The drug tolerance was determined by checking the CFU counts under the conditions seen with the two highest fluconazole concentrations. For spot assays, overnight cultures were adapted to an OD_600_ of 1, and 5 serial 1/5 dilutions were spotted. SDglu medium was used with different concentrations of fluconazole and doxycycline. The plates were incubated at 30°C for 48 or 72 h. All experiments were conducted with at least three biological repeats, and representative results are shown.

### Sterol measurement.

Sterols were extracted according to the method described in reference 77, with a few adaptations. In summary, cells were grown for 24 h in minimal medium, with or without 20 μg/ml fluconazole. The cells were collected, resuspended in saponification medium, and subjected to vortex mixing. The samples were incubated for 1 h at 80°C, after which 1 ml of water and 4 ml of hexane were added. After mixing, the two layers were allowed to separate. For spectrophotometrical analysis, UV-transmittable 96-well microtiter plates (3635; Costar Corning) were used to allow measurement of the OD_281_ and OD_230_. A formula from reference 38 was used to measure the percentages of ergosterol (corrected for cellular wet weight and resuspension volume). For GC-MS analysis, the sterols were extracted twice with hexane, which was then evaporated by vacuum centrifugation. The sterols were resuspended in 100 μl silylating mixture (85432; Sigma) and incubated at room temperature for 30 min. Finally, 500 μl hexane was added and the samples were immediately stored at −20°C for later analysis by GC-MS. One microliter of the sample was injected into a gas chromatograph-mass spectrometer (Shimadzu QP2010 Ultra Plus) equipped with an HP-5ms nonpolar column (Agilent) (30 m in length, 0.25-mm inner diameter [id.]; 0.25-µm thin layer). Helium was used as carrier gas with a flow rate of 1.4 ml/min. Injection was carried out at 250°C in split mode after 1 min and with a ratio of 1:10. The temperature was first held at 50°C for 1 min and then allowed to rise to 260°C at a rate of 50°C/min, followed by a second ramp of 2°C/min until 325°C was reached; that temperature was maintained for 3 min. The mass detector was operated in scan mode (50 to 600 atomic mass units [amu]), using electron impact ionization (70 eV). The temperatures of the interface and detector were 290°C and 250°C, respectively. A mix of linear *n*-alkanes (from C_8_ to C_40_) was injected to serve as external retention index markers. Sterols were identified by their retention time relative to the internal standard (cholestane) and specific mass spectrometric patterns using AMDIS version 2.71. The deconvoluted spectra were matched to GC-MS libraries described in reference 78 and NIST/EPA/NIH version 2011. Analysis was performed by integration over the base ion of each sterol, and abundance was calculated relative to the internal standard, comparing the relative peak areas of the compounds across treatments using two-way ANOVA with Bonferroni correction. Apart from the *P* values for pairwise comparison, the *P* values for interaction between the two parameters are also described.

### RNA extraction and gene expression analysis by qRT-PCR.

*S. cerevisiae* strains were grown in SDglu medium at 30°C for 24 h, with or without 20 μg/ml fluconazole. *C. glabrata* and *C. albicans* cells were incubated for 8 h at 37°C or 30°C in RPMI medium, with or without 1 μg/ml fluconazole. Cells were washed with ice-cold water, resuspended in TRIzol (Thermo, Fisher), and broken using glass beads and a FastPrep machine (MP Biomedicals). RNA was extracted by respective addition of chloroform and isopropanol and washed three times with 70% ethanol. Equal amounts of RNA were treated with a DNase enzyme (New England Biolabs) and converted to cDNA (iScript cDNA synthesis kit; Bio-Rad). Real-time quantitative PCR (qPCR) reactions were conducted using GoTaq polymerase (Promega) and a StepOnePlus real-time PCR device (Thermo, Fisher). Data were analyzed using qBasePlus software (Biogazelle) (79). Further data analysis and statistics analysis of log_2_(Y) transformed expression values were performed with Graphpad Prism. Transformation of the data points was performed to enable the use of standard statistical methods. Graphs show the means of the transformed values, together with their SEM. The statistical method used is mentioned under each figure. Copy number analysis of the genomic DNA of transformants was performed by qPCR, as described above.

### Western blotting.

*S. cerevisiae* strains were grown in SDglu medium for 24 h at 30°C, with or without 20 μg/ml fluconazole. Cells were washed with lysis buffer (200 mM sorbitol, 20 mM HEPES–KOH [pH 6.8], 1 mM EDTA, 50 mM potassium acetate and protease inhibitors [Roche]), and glass beads were added to break them using a FastPrep machine. The amount of proteins was quantified using the Pierce protein assay (Thermo, Fisher), and 6 μg was loaded per well on an Invitrogen NuPage Novex bis-Tris gradient gel (4% to 12%). We used anti-HA (12013819001; Roche) and anti-Pgk1 (459250; Invitrogen) as loading controls. The blots were visualized using a FujiFilm LAS-4000 mini system and accompanying software.

### Fluorescence microscopy.

To determine the location of Mge1-GFP inside the cell, we used a FluoView FV1000 confocal microscope (Olympus IX81) and its software. We visualized GFP with a 488-nm argon laser and BA505-540 emission filter and mCherry with a 559-nm laser and BA575-675 emission filter. A 60× UPlanSApo (numerical aperture [NA], 1.35) objective lens was used.
