# Supplementary figures and images for: Mitochondrial Cochaperone Mge1 Is Involved in Regulating Susceptibility to Fluconazole in Saccharomyces cerevisiae and Candida Species
Source: mBio. 2017 Jul 18;8(4):e00201-17. doi: 10.1128/mBio.00201-17 (PMC5516249; doi:10.1128/mBio.00201-17)

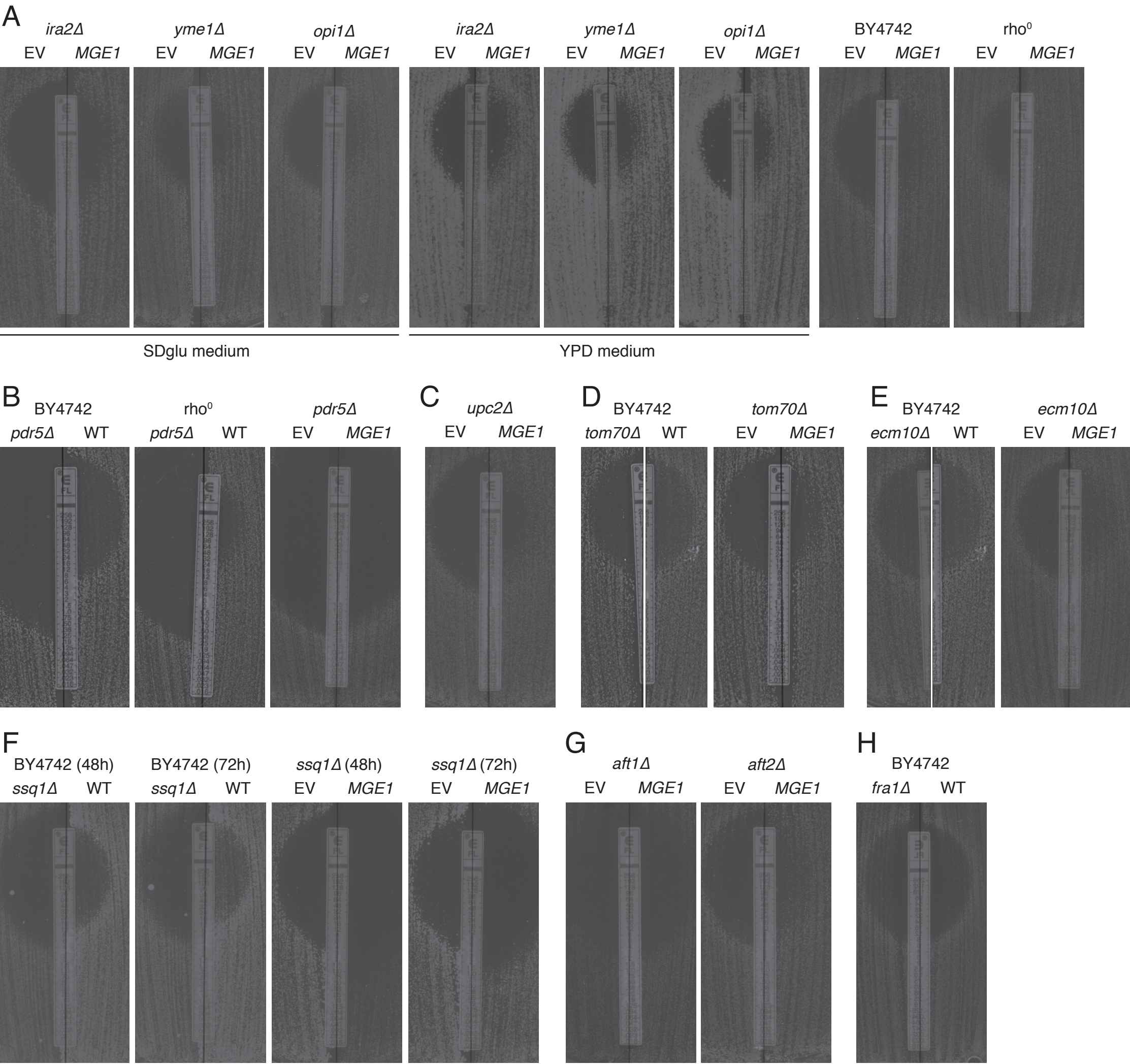

Supplement: FIG S1 [file mbo004173389sf1.pdf]

**A**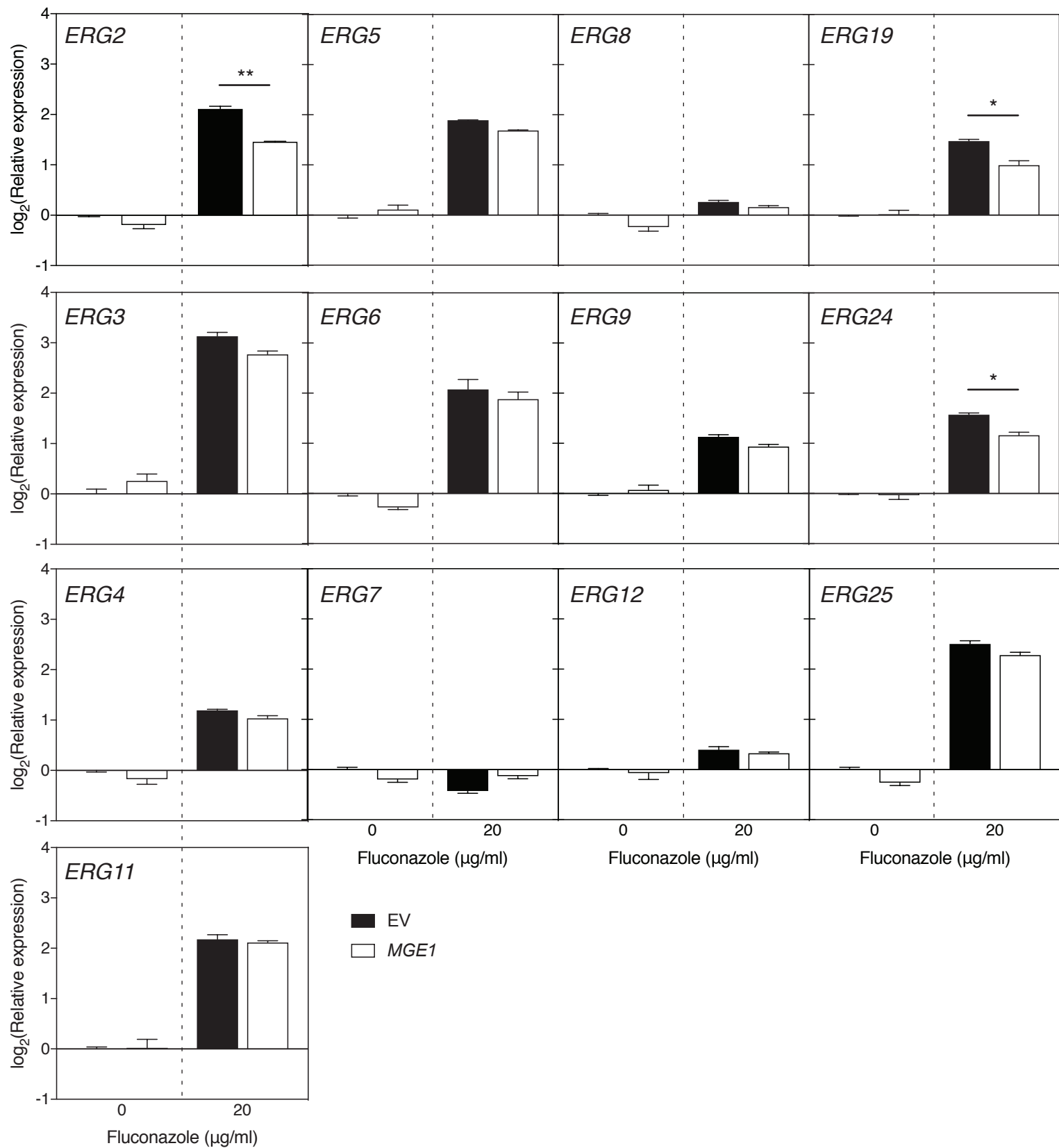**B**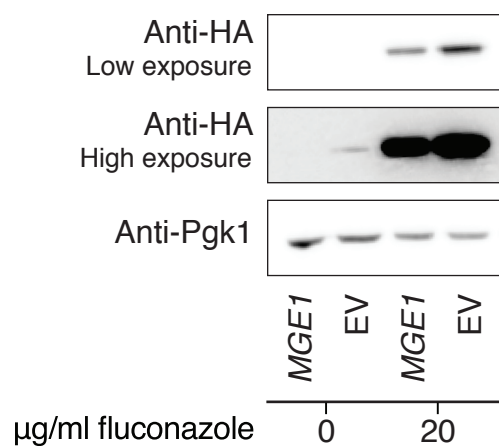**C**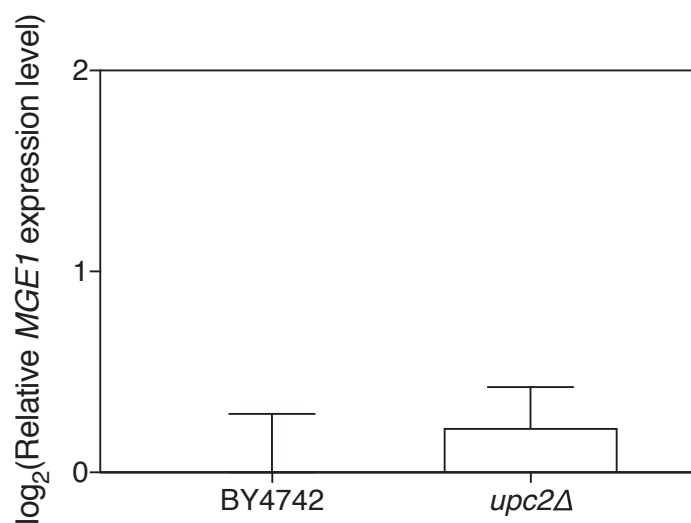

Supplement: FIG S3 [file mbo004173389sf3.pdf]

**A**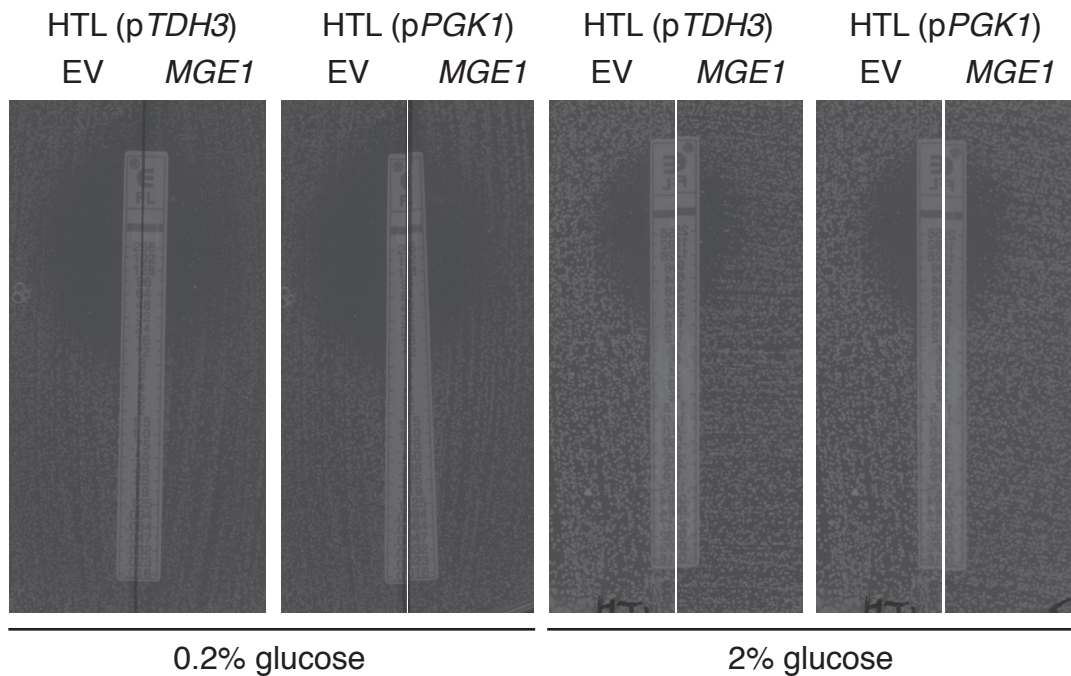**B**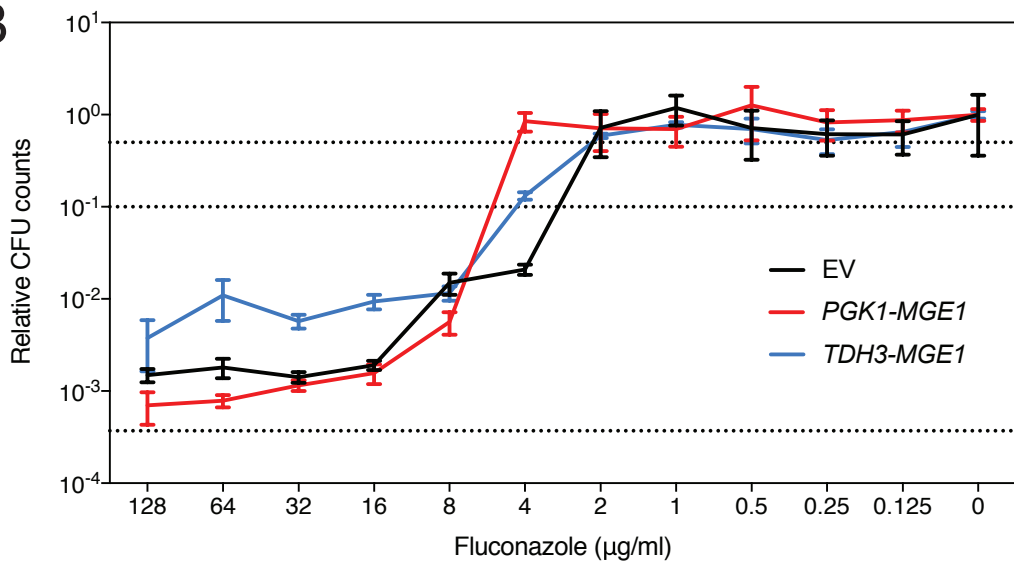

Supplement: FIG S4 [file mbo004173389sf4.pdf]
