# Supplementary material for: Mitochondrial Cochaperone Mge1 Is Involved in Regulating Susceptibility to Fluconazole in Saccharomyces cerevisiae and Candida Species
Source: mBio. 2017 Jul 18;8(4):e00201-17. doi: 10.1128/mBio.00201-17 (PMC5516249; doi:10.1128/mBio.00201-17)

A

| IUPAC Name | Trivial name | RT | RRT | Molecular ion TMS-derivative (g/mol) | Ion used for quantification (m/z) | Relative abundance | | | | | | | |
|---|---|---|---|---|---|---|---|---|---|---|---|---|---|
| | | | | | | 0 µg/ml fluconazole | | | | 20 µg/ml fluconazole | | | |
| | | | | | | EV | | *MGE1* | | EV | | *MGE1* | |
| | | | | | | Mean | SEM | Mean | SEM | Mean | SEM | Mean | SEM |
| (6E,10E,14E,18E)-2,6,10,15,19,23-Hexamethyl-tetracosa-2,6,10,14,18,22-hexaene | Squalene | 11,04 | 0,94 | (-) | 69 | 3,07 | 0,11 | 3,58 | 0,09 | 3,01 | 0,07 | 3,89 | 0,18 |
| Ergosta-5,7,22-trien-3ß-ol | Ergosterol | 17,11 | 1,46 | 468,90 | 363 | 1,28 | 0,06 | 1,65 | 0,06 | 0,32 | 0,01 | 0,96 | 0,05 |
| Ergosta-7-en-3ß-ol | / | 18,54 | 1,58 | 472,90 | 255 | 0,0343 | 0,0017 | 0,0275 | 0,0013 | 0,0011 | 0,0001 | 0,0058 | 0,0005 |
| 4,4,14-Trimethylcholesta-8,24(28)-dien-3ß-ol | Lanosterol | 19,04 | 1,63 | 498,90 | 393 | 0,46 | 0,02 | 0,65 | 0,02 | 1,29 | 0,03 | 0,84 | 0,05 |
| 14-Methylergosta-8,24(28)-dien-3ß-ol | 14-Methylfecosterol | 18,01 | 1,54 | 484,90 | 379 | 0,010 | 0,000 | 0,009 | 0,001 | 0,173 | 0,001 | 0,066 | 0,005 |
| 14-Methylergosta-8,24(28)-dien-3ß,6α-diol | / | 18,89 | 1,61 | 573,10 | 467 | 0,00020 | 0,00005 | 0,00107 | 0,00009 | 1,03 | 0,02 | 0,29 | 0,02 |
| Cholesta-8,24-dien-3ß-ol | Zymosterol | 16,73 | 1,43 | 456,90 | 69 | 0,017 | 0,001 | 0,021 | 0,001 | 0,0006 | 0,0001 | 0,0020 | 0,0001 |
| 4,4-Dimethylcholesta-8,24-dien-3ß-ol | T-MAS | 19,39 | 1,66 | 484,90 | 135 | 0,0345 | 0,0009 | 0,080 | 0,002 | 0,0015 | 0,0002 | 0,0089 | 0,0007 |
| Sterol X | | 16,37 | 1,40 | | 251 | 0,0432 | 0,0003 | 0,038 | 0,002 | 0,012 | 0,001 | 0,031 | 0,003 |
| Sterol Y | | 16,62 | 1,42 | | 123 | 0,0023 | 0,0016 | 0,0004 | 0,0001 | 0,134 | 0,004 | 0,029 | 0,003 |
| Sterol Z | | 17,57 | 1,50 | | 469 | 0,0013 | 0,0001 | 0,0019 | 0,0001 | 0,141 | 0,002 | 0,040 | 0,003 |

B

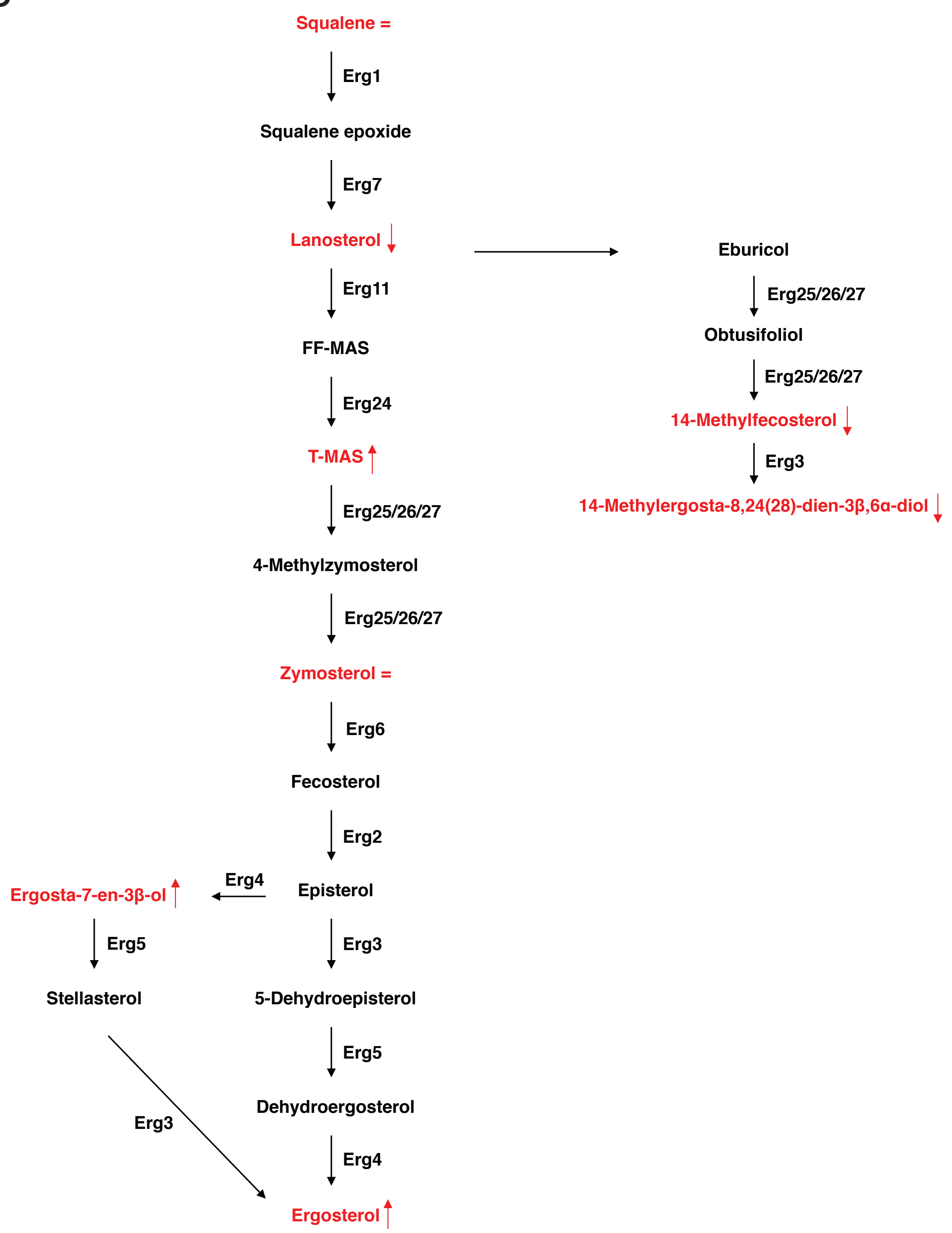

Supplement: FIG S2 [file mbo004173389sf2.pdf]
