# Supplementary material for: Mitochondrial Cochaperone Mge1 Is Involved in Regulating Susceptibility to Fluconazole in Saccharomyces cerevisiae and Candida Species
Source: mBio. 2017 Jul 18;8(4):e00201-17. doi: 10.1128/mBio.00201-17 (PMC5516249; doi:10.1128/mBio.00201-17)

A

Transformants	Copy number	Relative expression (with flu)	MIC_{flu}
LDa01	5,12	10,76	Higher
LDa02	3,13	6,60	Wild type
LDa03	3,22	4,80	Wild type
LDa04	3,11	3,99	Wild type
LDa05	3,07	17,43	Higher
LDa06	2,50	1,65	Wild type
LDa07	7,06	54,34	Higher
LDa08	3,96	18,69	Higher
LDa09 (EV)	2,00	1,00	Wild type

B

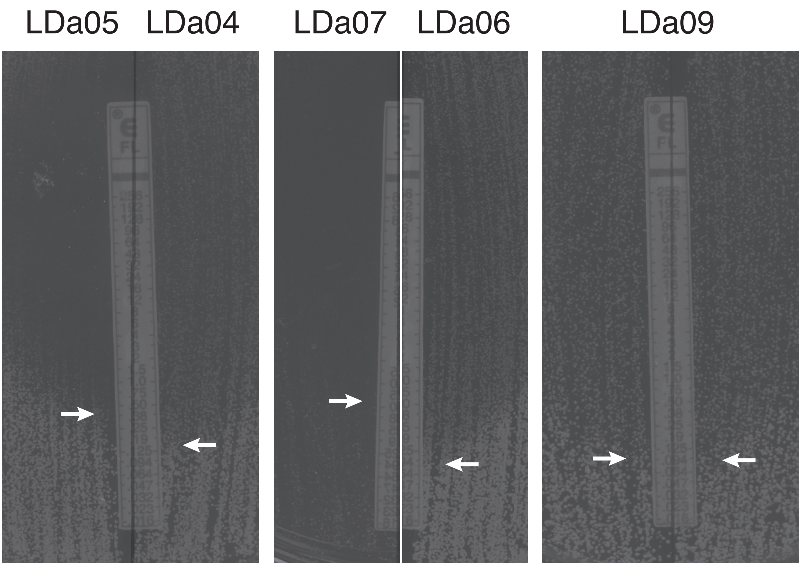

C

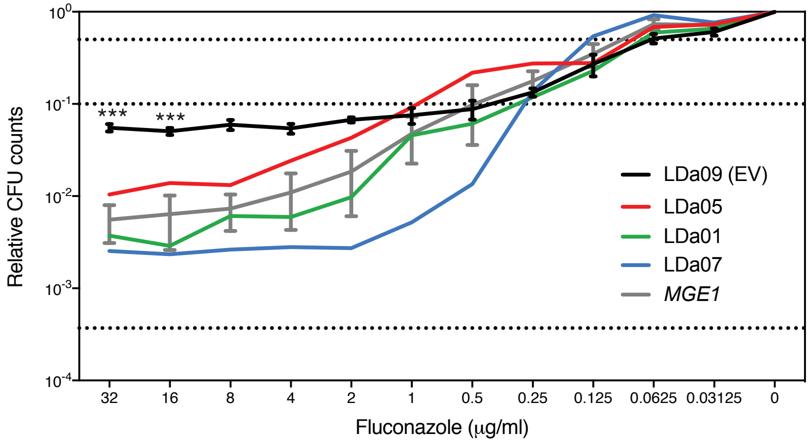

Supplement: FIG S5 [file mbo004173389sf5.pdf]
