# Supplementary material for: Mitochondrial Cochaperone Mge1 Is Involved in Regulating Susceptibility to Fluconazole in Saccharomyces cerevisiae and Candida Species
Source: mBio. 2017 Jul 18;8(4):e00201-17. doi: 10.1128/mBio.00201-17 (PMC5516249; doi:10.1128/mBio.00201-17)
Supplement: TABLE S1 [file mbo004173389st1.pdf]

Strain name	Genotype	Reference
Saccharomyces cerevisiae **strains**		
BY4742	*MATα; his3Δ; leu2Δ; lys2Δ; ura3Δ*	Euroscarf
AFc202	Same as BY4742 with *ERG11*-3xHA	This study
ira2Δ	Same as BY4742 with *ira2*Δ::*kanMX4*	Y.K.O. collection
opi1Δ	Same as BY4742 with *opi1*Δ::*kanMX4*	Y.K.O. collection
yme1Δ	Same as BY4742 with *yme1*Δ::*kanMX4*	Y.K.O. collection
pdr5Δ	Same as BY4742 with *pdr5*Δ::*hphNT1*	This study
*ssq1*Δ	Same as BY4742 with *ssq1*Δ::*kanMX4*	Y.K.O. collection
tom70Δ	Same as BY4742 with *tom70*Δ::*kanMX4*	Y.K.O. collection
ecm10Δ	Same as BY4742 with *ecm10*Δ::*kanMX4*	Y.K.O. collection
aft1Δ	Same as BY4742 with *aft1*Δ::*kanMX4*	Y.K.O. collection
aft2Δ	Same as BY4742 with *aft2*Δ::*kanMX4*	Y.K.O. collection
fra1Δ	Same as BY4742 with *fra1*Δ::*kanMX4*	Y.K.O. collection
rho^0	Same as BY4742, loss of mitochondrial DNA	This study
rho^0 *pdr5Δ*	Same as rho^0 with *pdr5*Δ::*hphNT1*	This study
Candida glabrata **strains**		
2001HTL	ATCC 2001 with *his3Δ; leu2Δ; trp1Δ*	(1)
Candida albicans **strains**		
SC5314	Clinical isolate, wild type	(2)
LDa01 to 08	Same as SC5314 with pLD01 integrated, Eight independent transformants with varying expression levels of *CaMGE1*	This study
LDa09	Same as SC5314 with CIp10-*NAT1* integrated	This study

Table S1. Strains used in this study.

1. **Schwarzmuller T, Ma B, Hiller E, Istel F, Tscherner M, Brunke S, Ames L, Firon A, Green B, Cabral V, Marcet-Houben M, Jacobsen ID, Quintin J, Seider K, Frohner I, Glaser W, Jungwirth H, Bachellier-Bassi S, Chauvel M, Zeidler U, Ferrandon D, Gabaldon T, Hube B, d'Enfert C, Rupp S, Cormack B, Haynes K, Kuchler K.** 2014. Systematic phenotyping of a large-scale *Candida glabrata* deletion collection reveals novel antifungal tolerance genes. PLoS Pathog **10:**e1004211.
2. **Fonzi WA, Irwin MY.** 1993. Isogenic strain construction and gene mapping in *Candida albicans*. Genetics **134:**717-728.
