# Supplementary material for: Mitochondrial Cochaperone Mge1 Is Involved in Regulating Susceptibility to Fluconazole in Saccharomyces cerevisiae and Candida Species
Source: mBio. 2017 Jul 18;8(4):e00201-17. doi: 10.1128/mBio.00201-17 (PMC5516249; doi:10.1128/mBio.00201-17)
Supplement: TABLE S2 [file mbo004173389st2.pdf]

Primer name	Sequence (5’->3’)
**Primers used for tagging *ScERG11* with 3xHA tag in the genome**	
ScERG11_3xHAtag_Fw	CAAGATCATCTGGGAAAAGAGAAATCCAGAACAAAAGATCAGGGAACAAAAGCTGG
ScERG11_3xHAtag_Rev	TCTCTTTTTCTGTTTTTTTTTTTTTCTCAGTTACAAACCCTATAGGGCGAATTGG
**Primers used for deleting *PDR5* in the genome of *S. cerevisiae* using *hphNT1***	
hphNT1_PDR5_Fw	ACTTTCGAAAAAGAAATTAAAGACCCTTTTAAGTTTTCGTATCCGCTCGTTCGAAAGACTTTAGACAAAACGTACGCTGCAGGTCGAC
hphNT1_PDR5_Rev	GTGATTATGTATGTTTATTAAAAAAGTCCATCTTGGTAAGTTTCTTTTCTTAACCAAATTCAAAATTCTAATCGATGAATTCGAGCTCG
**Primers used for cloning *MGE1-GFP* in YEPlac195**	
MGE1-GFP-Fw1	TCACGACGTTGTAAAACGACGGCCAGTGAATTCGAGCTCGGTACCTTCAGTGATATTTTAATACCTCGC
MGE1-GFP-Rev1	GTGAAAAGTTCTTCTCCTTTTGAACCACCTCCACCAGAACCTCCACCTCCGTTCTCTTCGCCCTTAAC
MGE1-GFP-Fw2	TTGTTAAGGGCGAAGAGAACGGAGGTGGAGGTTCTGGTGGAGGTGGTTCAAAAGGAGAAGAACTTTTCACTG
MGE1-GFP-Rev2	TATAGATAAAGTCGTAGGTAGTAATAATTCTTCATCAAGGCTATTTGTATAGTTCATCCATGCC
MGE1-GFP-Fw3	AGCTGCTGGGATTACACATGGCATGGATGAACTATACAAATAGCCTTGATGAAGAATTATTACTACC
MGE1-GFP-Rev3	TCACACAGGAAACAGCTATGACCATGATTACGCCAAGCTTGCATGCCTCGAGAAAATAAAAGGTG
**Primers used for cloning the *CaACT1* promoter, *NAT1* and *CaACT1* terminator in CIp10**	
NAT1_Cloning_Fw	AGATCAGCGGCCGCGTTTATAATAAACTTAGTC
NAT1_Cloning_Rev	TCGCCTACTAGTATTTTATGATGGAATGAATGGGAT
**Primers used for cloning the *CaACT1* promoter, *CaMGE1* and *CaACT1* terminator in the MCS of CIp10**	

pACT1_Cloning_Fw	TCGGTACGCGTCAGCCTCGTTTATAATAAACTTAG TCTAATATTTCTTTTGG
pACT1_Cloning_Rev	GCTCACTGCAGCATTTTGAATGATTATATTTTTTA ATATTAATATCGAG
CaMGE1_Cloning_Fw	AAACTGCAGATGCATAGAGCTTTATATAAT
CaMGE1_Cloning_Rev	CCATCGATTCAATTGTCATCTTCAGATTTG
tACT1_Cloning_Fw	GATACCTCGAGGAGTGAAATTCTGGAAATCTGGA AATCTGGTTTTGTATTC
tACT1_Cloning_Rev	CCAATGGTACCGACATTTTATGATGGAATGAATGG GATGAATCATCAAAC

Primers used for cloning *CgPGK1* or *CgTDH3* promoters and *CgMGE1* (with terminator) in pCgACH plasmid

pPGK1_Cloning_Fw	CGACGGCCAGTGAATTCGAGCTCCCGCGGAGACG GTTAGCCATCATATACGCATATACGA
pPGK1_Cloning_Rev	CTCTCATGTCGACTATCGAATAGATGTATGTATGC CGTCTTGCCAATTGACAACTAATCA
pTDH3_Cloning_Fw	CGACGGCCAGTGAATTCGAGCTCCCGCGGCGTCA TGCAGGGATTGTCCGTCTCC
pTDH3_Cloning_Rev	CTCTCATGTCGACTTTTGATGTTATGTTTGTTGTGA TTTGTAGGTGTTTTAATT
CgMGE1_Cloning pPGK1_Fw	ATCTATTCGATAGTCGACATGAGAGCTTTCAGTAA CGTTTCCAGAAT
CgMGE1_Cloning pTDH3_Fw	ATAACATCAAAAGTCGACATGAGAGCTTTCAGTA ACGTTTCCAGAAT
CgMGE1_Cloning_Rev	TTACGCCAAGCTTGCATGCGCGGCCGCTTATATAT ATCATTAGTATTATATATTTGAAACTGGTCTTTTG ACA

Primers used for qRT-PCR

ScMGE1_qPCR_Fw	ACAGGGGTTAGAATGACAAGAGA
ScMGE1_qPCR_Rev	CGTTGCTTCGTGTTTATTTGG
ScERG11_qPCR_Fw	CACGAATTTGTCTTCAACGCTAA
ScERG11_qPCR_Rev	AGTCAAATGAGCGTAAGCAGCTT
ScARN1_qPCR_Fw	TTGAAGGGCCAGAAGTCCTA

ScARN1_qPCR_Rev	AACCCATCAAAAGGACACCA
ScFET3_qPCR_Fw	CGGTGTGAATTACGCCTTCT
ScFET3_qPCR_Rev	TTTCGGAGTTGTTTGCTTGA
ScFIT1_qPCR_Fw	TTCTAGGGATGCCCAATCTG
ScFIT1_qPCR_Rev	GGATACCATTTTTGTGCTTGC
ScFRE1_qPCR_Fw	CACCAAAAAGTGCATGGTTCT
ScFRE1_qPCR_Rev	TCTGGATTGTTTGGATCACG
ScFTR1_qPCR_Fw	AAGAAAGGCGTGGTCACTTG
ScFTR1_qPCR_Rev	TTCCGTCAACTCCTGCTTTT
ScHMX1_qPCR_Fw	AACACGCTTGAAATGGGAAT
ScHMX1_qPCR_Rev	TCTGCGACGCTAATGATCTG
ScERG19_qPCR_Fw	GCGTATCATCAGTTGGTGCC
ScERG19_qPCR_Rev	AACACAGCATTTGGACCTGC
ScERG24_qPCR_Fw	CAAGCGCGTTACTTGAGTGT
ScERG24_qPCR_Rev	GCCGAGTGGAAGATGTGGAA
ScERG2_qPCR_Fw	TTGCCTCTCATTACGGGGAC
ScERG2_qPCR_Rev	GATCATTTGGCCCATCGCAC
ScERG5_qPCR_Fw	AACTTTGCGTTACAGACCTCCT
ScERG5_qPCR_Rev	GCCCTTTGGTGCGGTATAGTT
ScERG7_qPCR_Fw	CATTACACACCGTGGGGGAG
ScERG7_qPCR_Rev	CCAACCGCCATCCTTCATCT
ScERG25_qPCR_Fw	CATTACTGGGCTCACCGTCT
ScERG25_qPCR_Rev	AAGACCGAATGGAGCAGCG
ScERG6_qPCR_Fw	AGCATTGAAGAACTGTGGTTTCG
ScERG6_qPCR_Rev	AACGTACTTCCACTCACCAGTTA
ScERG3_qPCR_Fw	GTCTACCGTGCTCTGCACAA
ScERG3_qPCR_Rev	GCAAAAACCCGTCTACAGGA
ScERG4_qPCR_Fw	GCGAACGCATGTGCTAAAGG
ScERG4_qPCR_Rev	GTGTATGGGACACCGGCAAT
ScERG8_qPCR_Fw	ATCGAACCTCCCGTACAAACT
ScERG8_qPCR_Rev	GCGTCATAACCACCAGCAC
ScERG9_qPCR_Fw	CGGTATCCACGAGCAATCCA
ScERG9_qPCR_Rev	TGTAGCACTTCACGGTTGTTG

ScERG12_qPCR_Fw	TAAAGGCACCGATGACGAGG
ScERG12_qPCR_Rev	CCGATTGAGACAAGCAGTCCA
ScPDR5_qPCR_Fw	TGGGTCTGCTTGTCATTTCA
ScPDR5_qPCR_Rev	TGGCACTTGGGGTAGTCATAA
ScALG9_qPCR_Ref_Fw	CACGGATAGTGGCTTTGGTGAACAATTAC
ScALG9_qPCR_Ref_Rev	TATGATTATCTGGCAGCAGGAAAGAACTTGGG
ScSCR1_qPCR_Ref_Fw	TGGGATGGGATACGTTGAGAA
ScSCR1_qPCR_Ref_Rev	CTAGCCGCGAGGAAGGATT
Sc18S_qPCR_Ref_Fw	CACTTCTTAGAGGGACTATCGGTTTC
Sc18S_qPCR_Ref_Rev	CAGAACGTCTAAGGGCATCACA
CgMGE1_qPCR_Fw	GACGTTGAGAAGGCCAAGAG
CgMGE1_qPCR_Rev	CGCATGTCCAAAGTTATCCA
CgRPL10_qPCR_Ref_Fw	GAGATTCTTTCCACTTGAGAGTCAGA
CgRPL10_qPCR_Ref_Rev	CTCTCATACCTTGTTGCAATCTATCC
CgRPL13A_qPCR_Ref_Fw	ACCAAGCTGGTAAGAAGGAATCC
CgRPL13A_qPCR_Ref_Rev	GGAGCTCTAACGACTGGTCTCAA
CgRPL2A_qPCR_Ref_Fw	GCCGGTAAGAAGGCCTCTTT
CgRPL2A_qPCR_Ref_Rev	CTGTCACCTGGCTTTTCTTCAA
CgUBC13_qPCR_Ref_Fw	TGCCCGAGGACTACCCTATG
CgUBC13_qPCR_Ref_Rev	AGCACGTCCAGGCAGATACG
CaMGE1_qPCR_Fw	CCGCCATTTACAAGAAACTACC
CaMGE1_qPCR_Rev	CGTGACCAAGTGCCAAGTT
CaTEF1_qPCR_Ref_Fw	CCACTGAAGTCAAGTCCGTTGA
CaTEF1_qPCR_Ref_Rev	CACCTTCAGCCAATTGTTCGT
Ca18S_qPCR_Ref_Fw	GATGCCCTTAGACGTTCTGG
Ca18S_qPCR_Ref_Rev	CACGACGGAGTTTCACAAGA

Table S2. Primers used in this study.
