# Supplementary material for: Mitochondrial Cochaperone Mge1 Is Involved in Regulating Susceptibility to Fluconazole in Saccharomyces cerevisiae and Candida Species
Source: mBio. 2017 Jul 18;8(4):e00201-17. doi: 10.1128/mBio.00201-17 (PMC5516249; doi:10.1128/mBio.00201-17)
Supplement: TABLE S3 [file mbo004173389st3.pdf]

Plasmid	Backbone	Insert	Marker	Reference
pAFc86	YEPlac195	p*MGE1-ScMGE1*-t*MGE1*	*ScURA3*	This study
pESc01	YEPlac195	p*MGE1-ScMGE1-GFP*-t*MGE1*	*ScURA3*	This study
pESg01	pCgACH	p*PGK1-CgMGE1*-t*MGE1*	*CgHIS3*	This study
pESg02	pCgACH	p*TDH3-CgMGE1*-t*MGE1*	*CgHIS3*	This study
FBp459	pHS12	p*ADH1-preCox4-mCherry*	*ScLEU2*	(1)
CIp10-*NAT1*	CIp10	p*ACT1-CaNAT1*-t*ACT1*<br>p*ACT1*-t*ACT1* in MCS	*CaURA3*	This study
pLDa01	CIp10-*NAT1*	p*ACT1-CaMGE1*-t*ACT1*	*CaNAT1*	This study

Table S3. Plasmids used in this study.

1. **Swinnen E, Wilms T, Idkowiak-Baldys J, Smets B, De Snijder P, Accardo S, Ghillebert R, Thevissen K, Cammue B, De Vos D, Bielawski J, Hannun YA, Winderickx J.** 2014. The protein kinase Sch9 is a key regulator of sphingolipid metabolism in *Saccharomyces cerevisiae*. Mol Biol Cell **25:**196-211.
