# Supplementary material for: Mitochondrial Cochaperone Mge1 Is Involved in Regulating Susceptibility to Fluconazole in Saccharomyces cerevisiae and Candida Species
Source: mBio. 2017 Jul 18;8(4):e00201-17. doi: 10.1128/mBio.00201-17 (PMC5516249; doi:10.1128/mBio.00201-17)
Supplement: TABLE S4 [file mbo004173389st4.pdf]

Strain	MIC$_{flu}$ (µg/ml)		
		Broth microdilution assay	
	E-test	MIC$_{50}$	MIC$_{90}$
BY4742	6-8	8-16	16-32
rho^0	24-32	16-32	16-32
pdr5Δ	0,25	0,5-1	0,5-1
rho^0 *pdr5Δ*	0,25	0,5-1	0,5-1
tom70Δ	6-8	8-16	8-16
ecm10Δ	6-8	8-16	16-32
MGE1-GFP[a]	16	16-32	32-64
ssq1Δ	2-4[b]	0,25-0,5[b]	1-2[b]
AFc202	6	8-16	8-16
AFc202 EV	6	4-8	4-8
AFc202 *MGE1*	24-32	8-16	16-32
**Petite-negative strains in YPD**			
BY4742	12-16	8-16	8-16
BY4742 EV	16	8-16	16
BY4742 *MGE1*	64	16-32	16-32
ira2Δ EV	8-12	4-8	4-8
ira2Δ MGE1	32	8-16	8-16
yme1Δ EV	12-16	8-16	8-16
yme1Δ MGE1	48-64	16-32	16-32
opi1Δ EV	8-12	8-16	8-16
opi1Δ MGE1	48-64	8-16	16-32

Table S4. MIC$_{flu}$ values of several strains. [a] Overexpression of *MGE1-GFP*. [b] Determined after 72 hrs, on SCglu (latter only for E-test, BY4742 has MIC$_{flu}$ of 8-12 on this medium).
